# Safety and efficacy with esketamine in treatment-resistant depression: long-term extension study

**DOI:** 10.1093/ijnp/pyaf027

**Published:** 2025-05-04

**Authors:** Naim Zaki, Li (Nancy) Chen, Rosanne Lane, Teodora Doherty, Wayne C Drevets, Randall L Morrison, Gerard Sanacora, Samuel T Wilkinson, Allan H Young, Acioly L T Lacerda, Jong-Woo Paik, Vanina Popova, Dong-Jing Fu

**Affiliations:** Department of Neuroscience, Johnson & Johnson, Titusville, NJ, United States; Department of Clinical Biostatistics, Johnson & Johnson, Titusville, NJ, United States; Department of Clinical Biostatistics, Johnson & Johnson, Titusville, NJ, United States; Department of Neuroscience, Johnson & Johnson, Titusville, NJ, United States; Department of Neuroscience, Johnson & Johnson, San Diego, CA, United States; Department of Neuroscience, Johnson & Johnson, Titusville, NJ, United States; Department of Psychiatry, Yale University School of Medicine, New Haven, CT, United States; Department of Psychiatry, Yale University School of Medicine, New Haven, CT, United States; Institute of Psychiatry, Psychology and Neuroscience, King’s College, London, United Kingdom; Department of Psychiatry, Federal University of Sao Paulo, Sao Paulo, Brazil; Department of Psychiatry, Kyung Hee University, College of Medicine, Seoul, Republic of Korea; Department of Neuroscience, Johnson & Johnson, Beerse, Belgium; Department of Neuroscience, Johnson & Johnson, Titusville, NJ, United States

**Keywords:** esketamine, treatment-resistant depression, long-term, safety, efficacy

## Abstract

**Importance:**

The rates of relapse and suicide risk are higher in treatment-resistant depression (TRD) vs non-treatment-resistant major depressive disorder. Even among patients with TRD who initially respond, the majority (70%) relapse within 6 months.

**Objective:**

To evaluate the long-term safety and efficacy of esketamine nasal spray, combined with an oral antidepressant, in patients with TRD.

**Design:**

Phase 3, open-label, single-arm long-term extension study (SUSTAIN-3) conducted from June 2016 to December 2022.

**Setting:**

Outpatient.

**Participants:**

Adults with TRD who participated in ≥1 of 6 phase 3 “parent” studies continued esketamine by either entering a 4-week induction phase followed by an optimization/maintenance phase of variable duration (*n* = 458) or directly entering the optimization/maintenance phase of SUSTAIN-3 (*n* = 690), based on their individual response to study drug at the endpoint of the parent study.

**Interventions:**

Intranasal esketamine dosing was flexible, twice-weekly during induction and individualized to depression severity during optimization/maintenance (weekly, every-other-week, or every-4-weeks), under direct supervision by site staff.

**Main Outcomes and Measures:**

To assess the long-term safety of esketamine. Efficacy endpoints included the change in depressive symptoms, assessed by the Montgomery-Åsberg Depression Rating Scale (MADRS).

**Results:**

A total of 1148 patients were enrolled. Total exposure to esketamine was 3777 cumulative patient-years. Mean (median, range) exposure to esketamine in SUSTAIN-3 was 42.9 (45.8, range 0-79) months. The most common adverse events were headache (36.9%), dizziness (33.9%), nausea (33.6%), dissociation (25.5%), nasopharyngitis (23.8%), somnolence (23.1%), dysgeusia (20.2%), and back pain (20.0%). During the study, 5.3% and 6.4% of participants discontinued due to lack of efficacy or adverse event, respectively. Nine participants died: COVID-19-related (*n* = 3), pneumonia (*n* = 2), and completed suicide, myocardial infarction, multiple injuries, unknown cause (*n* = 1 each). The mean MADRS total score decreased during induction, and this reduction persisted during optimization/maintenance (mean [SD] change from baseline-to-phase endpoint of each phase: induction: −12.8 [9.73]; optimization/maintenance: + 0.2 [9.93]). A total of 35.6% of participants were in remission at the induction endpoint, and 48.5% and 49.6% at week 112 and optimization/maintenance endpoint, respectively.

**Conclusions and Relevance:**

In the SUSTAIN-3 final dataset, no new safety signals were identified during long-term treatment with intermittently-dosed esketamine, combined with oral antidepressant, and improvement in depression generally persisted among participants who remained on maintenance treatment. These results add to the accumulated evidence on TRD treatment with esketamine.

**Trial Registration:**

clinicaltrials.gov identifier: NCT02782104.

**Significance Statement**

Given the high relapse rate reported in previous studies of patients with treatment-resistant depression (TRD), there is clinical interest in longer-term safety and efficacy data with esketamine. In SUSTAIN-3, an open-label single-arm study, 1148 adults with TRD received intermittently-dosed esketamine nasal spray plus oral antidepressant for up to 6.5 years (3777 cumulative patient-years). No new safety concerns were identified vs prior short- and long-term (~1 year) studies. Most adverse events were transient, occurring and resolving on a dosing day. Depressive symptoms improved during the 4-week induction phase; improvement generally persisted during the long-term optimization/maintenance phase. Few participants discontinued across phases due to lack of efficacy (5.3%) or adverse event (6.4%). Within the context of an open-label, single-arm design, the long-term safety data and durability of the treatment effect observed in SUSTAIN-3 add substantially to the body of evidence supporting the treatment of TRD using esketamine nasal spray.

## INTRODUCTION

Treatment-resistant depression (TRD) is associated with a higher rate of relapse and a higher risk of suicide compared to non-treatment-resistant major depressive disorder (MDD).^1,2^ Even among patients with TRD who initially respond to standard antidepressants, the majority (~70%) relapse within 6 months.^3^ The longer patients remain unsuccessfully treated, the worse their prognosis.^4^

The unmet need for new TRD treatment options with sustained efficacy and safety has been noted for decades. In this regard, esketamine nasal spray, in conjunction with an oral antidepressant, was approved for TRD by the United States Food and Drug Administration^5^ and the European Medicines Agency,^6^ followed by approvals from >75 health authorities worldwide. These approvals were based on phase 2/3 short-term studies of TRD patients^7-10^ as well as a relapse prevention study^11^ and an open-label, 1-year safety study.^12^

The SUSTAIN-3 trial (NCT02782104) was conducted to assess longer-term safety (including cognitive, hepatic, and renal safety) and sustained efficacy of esketamine nasal spray, combined with an oral antidepressant, in patients with TRD. Reported herein are the final results of SUSTAIN-3, which support and extend the findings from the previously published interim data analyses.^13^ In that manuscript, we presented evidence for safety and durability of treatment effect across a total of 2769 cumulative patient-years; the current manuscript presents data from an additional 1008 patient-years of treatment with esketamine nasal spray.

## METHODS

### Ethical Practices

The study protocol and its amendments were approved by the Institutional Review Board (United States) or Independent Ethics Committee (all other locations) at each site (Methods S1, online Supplementary Material). The study was conducted in accordance with the ethical principles of the Helsinki Declaration. All individuals provided written informed consent before entering the study.

### Study Design

SUSTAIN-3 (NCT02782104), a phase 3, open-label, single-arm, multicenter, long-term extension study, was conducted between June 2016 and December 2022 at 222 sites in 27 countries/locations. The study consisted of 2 phases: a 4-week induction phase (if applicable) and an optimization/maintenance phase of variable duration (these phases are defined under the Study Drug section below).

### Study Population

Participants in 6 phase 3 “parent” studies of esketamine enrolled into the 4-week induction phase or the long-term optimization/maintenance phase of SUSTAIN-3 based on their clinical status at the parent study end. Participants who did not meet the criteria for response at the parent study end enrolled into the induction phase of SUSTAIN-3, and responders at the end of induction progressed to the optimization/maintenance phase. Participants who met the criteria for response at the parent study end, or with the Sponsor’s approval, enrolled directly into the optimization/maintenance phase of SUSTAIN-3 (Figure S1).

Participants in esketamine parent studies were adults (≥18 years) with TRD, defined as non-response to ≥2 antidepressants in the current episode of depression, as assessed using the Massachusetts General Hospital Antidepressant Treatment Response Questionnaire. Other eligibility criteria of each parent study are reported elsewhere.^8-12,14^

### Study Drug

Dosing of esketamine nasal spray was flexible, with 3 doses available (28 mg [starting dose participants aged ≥65 years], 56 mg, or 84 mg). During the induction phase, participants self-administered esketamine twice-weekly for 4 weeks to induce the clinical response. During optimization/maintenance, interval dosing of esketamine was weekly, bi-weekly, or every-4-weeks to maintain the antidepressant response, individualized to participants’ depression severity (per a clinical global impression–severity^15^ [CGI-S]-based algorithm^13^; refer to Table S1). Esketamine dosing occurred only at clinical sites, directly supervised by site staff.

Participants were instructed to take a standard-of-care oral antidepressant daily (except monoamine oxidase inhibitor), determined per the study site investigator’s clinical judgment. Changes to the oral antidepressant medication(s) or add-on treatment with mood stabilizers or antipsychotics were also permitted.

### Safety and Efficacy Assessments

#### Safety

Adverse events and other safety assessments (ie, hematology, chemistry, urinalysis, vital signs, electrocardiogram) were monitored throughout the study. Adverse events of special interest included events such as blood pressure (BP) elevation and respiratory depression, which were monitored during each dosing visit using vital sign measures that included pulse oximetry; liver enzyme elevation via laboratory testing; and events related to renal disorder (cystitis, dysuria, pollakiuria, nephrolithiasis, micturition urgency, urinary incontinence, hematuria). Urine was screened every 12 weeks for illicit drugs (ie, barbiturates, methadone, opiates, cocaine, phencyclidine, amphetamine/methamphetamine).

The Columbia-Suicide Severity Rating Scale^16^ was used to evaluate potential suicidal ideation and behavior, the Modified Observer’s Assessment of Alertness/Sedation (MOAA/S) scale to evaluate postdose sedation, and the Clinical Global Assessment of Discharge Readiness (CGADR) to evaluate participants’ discharge readiness from study sites following dosing, based on their overall clinical status. Cognition was assessed using the Cogstate computerized test battery^17,18^ and the Hopkins Verbal Learning Test-Revised^19^. Descriptions of these safety measures appear in the online Supplementary Material (Methods S2).

#### Efficacy

The clinician-rated efficacy measures used to rate the severity of depressive symptoms and depressive illness, respectively, were the Montgomery-Åsberg Depression Rating Scale^20^ (MADRS) and the CGI-S.^15^ Patient-rated outcome measures used for participants to rate their depressive symptoms and socio-occupational disability, respectively, were the Patient Health Questionnaire 9-item^21^ (PHQ-9) and the Sheehan Disability Scale^22^ (SDS). Descriptions of these scales appear in the online Supplementary Material (Methods S2).

### Data Quality Assurance

The SUSTAIN-3 study was monitored according to the Sponsor’s current Standard Operating Procedure for the Monitoring of Clinical Trials. Steps taken to ensure the accuracy and reliability of the clinical study data included the selection of qualified investigators and appropriate study sites, review of protocol procedures with the investigator and associated study site personnel prior to study start, and periodic monitoring visits by the Sponsor or their delegate. Only qualified, trained, and certified raters could administer efficacy and safety scales.

### Statistical Methods

The number (percentage) of participants with adverse events, serious adverse events, and adverse events leading to discontinuation of the study drug were summarized by standardized Medical Dictionary for Regulatory Activities (MedDRA) preferred term, version 25.1. Study drug exposure was reported as cumulative person-years. The number of patients who attempted suicide, the number who died by suicide, and the number who died by any cause were each reported per 100 patient-years (exposure-adjusted rate), a standardized measure of risk, allowing comparison of the SUSTAIN-3 data to data from studies of other TRD treatments.

Analyses of cognitive data were conducted by age group (ie, <65 years old and ≥65 years old). Descriptive statistics, including means and mean change from baseline scores, were calculated for cognition tests; in addition, the difference in each participant’s performance on any cognitive measure from baseline of the optimization/maintenance phase was characterized based on the Reliable Change Index (RCI), with an absolute value of RCI ≥1.96 (ie, −1.96 indicates that test performance was worse than at baseline and 1.96, better than at baseline) considered to be a meaningful change from the baseline score for a test. Cohen’s d was calculated to further evaluate the magnitude of any cognitive changes. Descriptive statistics (mean and SD or SE) were provided for other safety parameters.

Efficacy data were summarized by descriptive statistics. Efficacy endpoints include: change from baseline in depressive symptoms (MADRS; PHQ-9); proportion of participants who achieved response (defined as ≥50% improvement from baseline) and remission (MADRS score ≤12; PHQ-9 score <5)^23^; overall severity of illness (CGI-S); and change from baseline in functioning and associated disability (SDS score); proportion of participants who achieved response (SDS scores ≤4 for each item, ≤12 for the total score) and remission (SDS ≤2 for each item score, ≤6 for the total score).^24^ In efficacy analyses for the *induction* phase, baseline was the last observation prior to or on the start date of the induction phase for participants who entered SUSTAIN-3 at the induction phase. In efficacy analyses for the *optimization/maintenance* phase, baseline was the last observation prior to or on the start date of the optimization/maintenance phase.

## RESULTS

**Figure 1.**
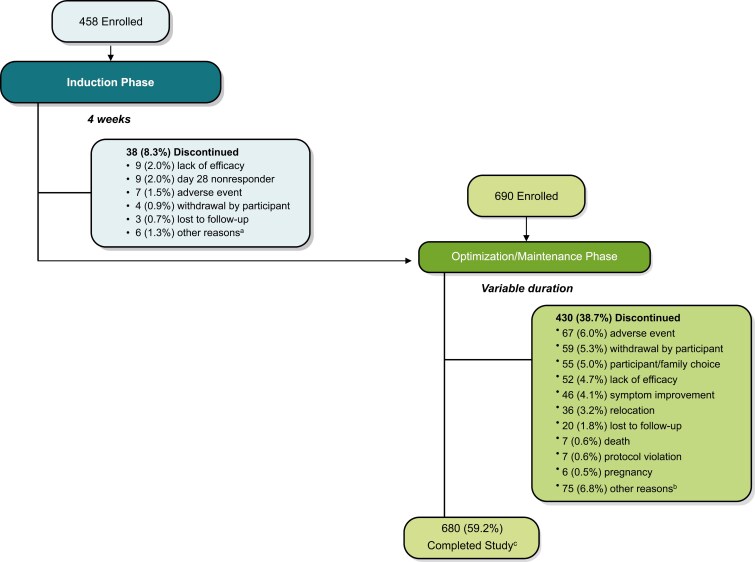
SUSTAIN-3 participant disposition. ^a^One each: scheduling conflicts; multiple reasons (mainly, feeling better, wanted to start working again, adverse events, and time requirements of study); withdrew consent; participant’s choice despite investigator’s advice to continue; relocation; and death on study day 26 due to completed suicide, 4 days after the last dose of esketamine. ^b^Other reasons (each ≤ 1%, eg, investigator/sponsor decision, employment/school, personal reasons). ^c^A participant was considered to have completed the study if they actively participated in the induction or optimization/maintenance phase until esketamine was approved in the respective country and accessible through the local healthcare system funding or until the end of December 2022, whichever was earlier. Note: Participants were eligible to enroll into the induction phase or the optimization/maintenance phase based on their status at the end of the parent study. Participants received open-label esketamine nasal spray (28 mg [only an option for participants ≥65 years], 56 mg, or 84 mg) twice per week during the induction phase, and weekly, every-other-week, or every 4 weeks, based on clinical global impression—severity (CGI-S) and tolerability, during the optimization/maintenance phase. Data for the induction phase are reported elsewhere.^13^

A total of 1148 adult patients with TRD were enrolled into SUSTAIN-3 (Figure 1). The number of participants enrolled into SUSTAIN-3 is summarized by parent study and entry point in Table S2. Participants’ mean (SD) age was 49.6 (12.28) years at baseline and two-thirds were female. Demographics at baseline are shown in Table 1.

**Table 1.** Demographics at baseline.

	Esketamine nasal spray *N* = 1148
Age (years)	
Mean (SD)	49.6 (12.28)
Range	19–83
≥65, *n* (%)	122 (10.6)
Sex, *n* (%)	
Male	384 (33.4)
Female	764 (66.6)
Race^a^, *n* (%)	
Asian	45 (3.9)
Black or African American	45 (3.9)
American Indian or Alaskan Native	1 (0.1)
White	996 (86.8)
Multiple	10 (0.9)
Other^b^	29 (2.5)
Not reported	22 (1.9)
Employment status^c^, *n* (%)	
Any type of employment	693 (60.4)
Any type of unemployment	281 (24.5)
Other	174 (15.2)
History of hypertension prior to study participation, *n* (%)	
Yes	271 (23.6)
No	877 (76.4)
Region, *n* (%)	
Europe	486 (42.3)
North America	343 (29.9)
Other	319 (27.8)

^a^According to participant self-report.

^b^Includes participants self-reported as half-blood (*n* = 12), mixed (*n* = 10), mixed origin (*n* = 4), Mulatto (*n* = 2), and mixed Black/White (*n* = 1).

^c^Any type of employment includes: any category containing “Employed” Sheltered Work, Housewife or Dependent Husband, and Student; any type of unemployment includes: any category containing “Unemployed”; Other includes: Retired and No Information Available.

Note: This table contains data that are reported elsewhere.^13^

Mean exposure to esketamine in SUSTAIN-3 was 42.9 months (median 45.8, range 0-79 months), with 728 (63.4%) treated for ≥36 months, 496 (43.2%) for ≥48 months, and 322 (28.0%) for ≥60 months (Figure S2); total exposure was 3777 cumulative patient-years. The mean (range) cumulative duration of intermittent esketamine treatment during the parent and SUSTAIN-3 studies combined was 48.3 (0-88) months, with 991 (86.3%) participants treated for ≥12 months, 867 (75.5%) for ≥24 months, 777 (67.7%) for ≥36 months, 582 (50.7%) for ≥48 months, 432 (37.6%) for ≥60 months, and 111 (9.7%) for ≥72 months; total exposure was 4247 cumulative patient-years.

During maintenance, most participants received 84 mg (64.4%) as their final esketamine dose, while 33.0% received 56 mg and 2.5% received 28 mg. The most frequent dosing interval was weekly (~40%-50% of participants for most of their dosing); every-other-week was ~35%-45%, and every-4-week dosing was ≤~25% (Figure S3).

The most common concomitant oral antidepressants taken by participants in the optimization/maintenance phase were duloxetine (36.7%), venlafaxine (27.7%), escitalopram (26.2%), sertraline (18.6%), and bupropion (11.2%) (Table S3). The most common antipsychotics used in the phase were quetiapine (7.4%) and aripiprazole (3.8%), and the most common mood stabilizer was lithium (2.3%).

### Safety

#### Treatment-Emergent Adverse Events

The most common adverse events (≥20%) were headache, dizziness, nausea, dissociation, nasopharyngitis, somnolence, dysgeusia, and back pain (Table 2). For adverse events occurring on an esketamine dosing day, most events (76769/79118 [97%]) resolved the same day.

**Table 2.** Most frequently (≥10%) reported adverse events in SUSTAIN-3.

	Esketamine nasal spray *N* = 1148
Participants with adverse event(s)	1089 (94.9%)
Most frequently reported adverse events:	
Headache	424 (36.9%)
Dizziness	389 (33.9%)
Nausea	386 (33.6%)
Dissociation	293 (25.5%)
Nasopharyngitis	273 (23.8%)
Somnolence	265 (23.1%)
Dysgeusia	232 (20.2%)
Back pain	230 (20.0%)
Anxiety	214 (18.6%)
Vertigo	213 (18.6%)
Arthralgia	188 (16.4%)
Diarrhea	188 (16.4%)
Vomiting	182 (15.9%)
Urinary tract infection	181 (15.8%)
Increased blood pressure	166 (14.5%)
Insomnia	162 (14.1%)
Fatigue	157 (13.7%)
Upper respiratory tract infection	145 (12.6%)
COVID-19	141 (12.3%)
Influenza	134 (11.7%)
Blurred vision	124 (10.8%)
Cough	118 (10.3%)
Hypoesthesia	116 (10.1%)

Serious adverse events were reported for 18.8% (216/1148) of participants (Table S4). Serious adverse events related to depression (MedDRA terms: depression, major depression, persistent depressive disorder, affect liability) occurred in 23 (2.0%) participants. Serious adverse events related to suicidality occurred in 28 (2.4%) participants, including suicide attempt (15, 1.3%), suicidal ideation (11, 1.0%), depression suicidal (1, 0.1%), and completed suicide (1, 0.1%). Of all serious adverse events, investigators considered one event (BP diastolic increased) to be very likely related, 4 events (arrhythmia, spontaneous abortion, hypertensive emergency, loss of consciousness) possibly related, and the remainder of events doubtfully or not related to esketamine. All 5 events considered very likely or possibly related to esketamine resolved (ie, participants recovered from the events without sequalae). There were 9 (0.8%) deaths, none considered related to esketamine (Results S1, online Supplementary Material).

Of the 1148 participants, 74 (6.4%) discontinued esketamine due to adverse events (Figure 1). The most common adverse events leading to discontinuation included: elevated BP (8 [0.7%]), including BP increased (*n* = 6), systolic BP increased (*n* = 1), and hypertensive emergency (*n* = 1), all in participants with hypertension history; (worsening) depression or major depression (*n* = 6 [0.5%]); and dissociation (*n* = 5 [0.4%]) (Table S5).

Dissociation, including reports of perceptual disturbances, derealization, or depersonalization, was reported as an adverse event during at least one dosing session for 25.5% of participants (Table 2), in 10.4% (365/3526) and 4.4% (5720/130976) of dosing sessions during the induction and optimization/maintenance phases, respectively. Almost all (99.8%) dissociation events occurred and resolved on the day of dosing across study phases. Thirty-six participants (3.1%) had a dissociation event(s) that persisted beyond 2 hours, the occurrence of which did not increase over long-term treatment (Figure S4). Few participants (*n* = 5) experienced a dissociation event that led to the withdrawal of the study drug (Table S5). There were no serious adverse events of dissociation.

Sedation was reported as an adverse event during at least one dosing session for 8.2% (94/1148) of participants, in 3.0% (107/3526) and 1.0% (1310/130976) of dosing sessions during the induction and optimization/maintenance phases, respectively. The majority (99.6%) of sedation events occurred on a dosing day and resolved the same day. There were no serious adverse events or adverse events of sedation that led to the withdrawal of the study drug.

The MOAA/S score decreased within 15 minutes of dosing, plateaued at 30 to 45 minutes, and returned to near predose levels by 1 hour during both the induction and optimization/maintenance phases. Clinically-relevant sedation, defined by MOAA/S score ≤3 (moderate sedation: responds after name called loudly or repeatedly) occurred at least once in 6.1% (28/458) of patients in the induction phase and 7.2% (80/1110) of patients in the optimization/maintenance phase.

No case of treatment-related interstitial/ulcerative cystitis was reported. Urinary tract infections were reported in 181 (15.8%) participants. Other adverse events (incidence ≥1%) related to a renal disorder included dysuria (3.0%), cystitis (2.4%), pollakiuria (2.4%), nephrolithiasis (1.7%), micturition urgency (1.5%), urinary incontinence (1.5%), and hematuria (1.3%).

A minority (7.9%) of participants experienced ≥1 hepatic adverse events, the most common being gamma-glutamyl transferase increased (2.5%), alanine aminotransferase increased (1.9%), hepatic enzyme increased (1.6%), hepatic steatosis (1.5%), cholelithiasis (1.4%), and aspartate aminotransferase increased (1.3%). The occurrence of hepatic adverse events did not increase over time (Figure S5). Few participants discontinued esketamine due to hepatic adverse events (Table S5).

#### Vital Signs

The greatest mean [SD] change in systolic BP and diastolic BP from predose to a scheduled postdose timepoint (*n* >10 participants with data) were all at the 40-minute postdose assessment (induction phase: + 9.5 [11.95] mmHg at day 15 and + 6.1 [8.35] mmHg at day 15, respectively; optimization/maintenance phase: + 8.8 [10.46]/+8.8 [10.63] mmHg at weeks 1/3 and + 5.6 [7.86]/+5.6 [7.38] mmHg at weeks 2/4, respectively). Mean systolic and diastolic BP values subsequently returned to predose values at the 1.5-hour postdose timepoint during dosing sessions. A minority (6.5%, 75/1148) of participants met study criteria for markedly elevated BP (ie, systolic BP ≥180 mmHg or diastolic BP ≥110 mmHg) at any time during the study. The rate was higher among participants with a history of hypertension compared with those without a history of hypertension (systolic BP ≥180 mmHg: 6.6% vs 1.5%; diastolic BP ≥110 mmHg: 9.2% vs 3.6%).

Investigators reported adverse events related to increased BP for 19.9% of participants, including BP increased (*n* = 166 [14.5%]), hypertension (79 [6.9%], all with history of hypertension), BP diastolic increased (24 [2.1%]), BP systolic increased (16 [1.4%]), and hypertensive emergency (1 [0.1%]). Incidence rates of increased BP events were generally similar at visits throughout both the induction and optimization/maintenance phases (Figure S6). An adverse event related to increased BP was reported in 3.6% (126/3526) and 1.0% (1312/130976) of dosing sessions during the induction and optimization/maintenance phases, respectively. Most (≥95%) increased BP events, including the event of hypertensive emergency, occurred and resolved on the day of dosing.

Postdose oxygen saturation (SpO2) levels <93% at ≥2 consecutive assessments were reported for a few participants (induction phase: 5/458 [1.1%]; optimization/maintenance phase: 9/1110 [0.8%], including 1 participant in both phases). Of all dosing sessions with postdose SpO_2_ measurements, 0.1% and <0.1% of dosing sessions in the induction and optimization/maintenance phases, respectively, had postdose SpO_2_ levels <93% at ≥2 consecutive assessments.

Four (0.3%) participants had oxygen saturation decreased reported as a treatment-emergent adverse event, none requiring concomitant treatment or intervention. These incidences were transient, asymptomatic, and resolved spontaneously in the postdose period, prior to discharge. No adverse events of respiratory depression or respiratory rate decreased were observed.

#### Discharge Readiness

Based on investigators’ assessments (CGADR), 89% of participants were ready to be discharged from clinic by 1.5 hours postdose on day 25 of the induction phase and 98% on week 276 of the optimization/maintenance phase.

#### Cognitive Effects

Cognition was assessed across multiple domains and remained stable, without clinically meaningful changes over time for the subgroup of participants <65 years of age (*N*~965) and the subgroup of participants ≥65 years of age (*N*~120) (online Supplementary Material, Results S2, Figures S7 through Figure S15). In participants ≥65 years, performance on tests of verbal and working memory and executive function remained stable or slightly improved based on arithmetic means and mean changes from baseline, while intraindividual variability increased (Figures S9 through Figure S15). Small increases in simple and choice reaction times occurred during the optimization/maintenance phase among both the participants <65 years as well as the older participants (Figures S7 and S8). Effect size (Cohen’s d) data for these 2 tests are presented in Table S6 and summarized in the online Supplementary Material, Results S2. Reliable Change Index scores for Detection and Identification are presented by age subgroup in Table S7.

#### Suicidal Ideation and Behavior

The Columbia-Suicide Severity Rating Scale scores over time are provided by phase in Figure S16. The percentage of participants reporting no events of suicidal ideation or behavior increased over time during the induction phase, from 79.9% at baseline to 93.2% at induction endpoint (Figure S16a). Throughout the optimization/maintenance phase, >90% of participants reported no events of suicidal ideation or behavior (Figure S16b).

### Efficacy

Efficacy results for the induction phase are reported elsewhere,^13^ but are also reported below (from the final dataset) to facilitate comparison to efficacy results for the optimization/maintenance phase.

#### Depressive Symptoms

**Figure 2.**
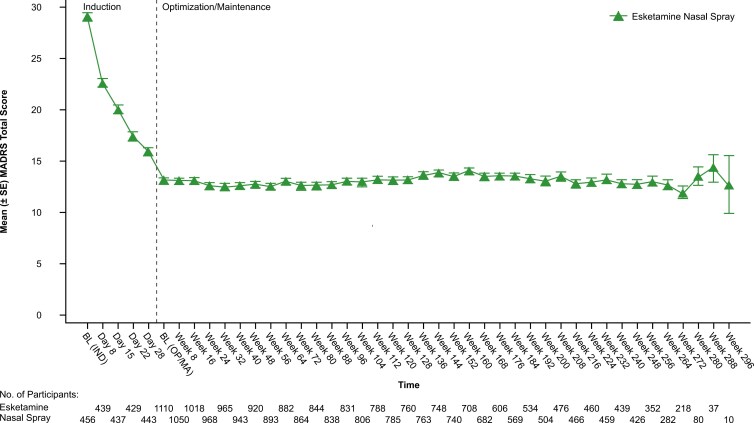
Mean (± SE) Montgomery-Åsberg Depression Rating Scale Total Score (Observed Cases). BL (IND), baseline (induction phase); BL (OP/MA), baseline (optimization/maintenance phase); MADRS, Montgomery-Åsberg Depression Rating Scale. Note: Responders from the induction phase of the SUSTAIN-3 study and responders from parent studies were to enter the optimization/maintenance phase. The greater variability of the mean MADRS score at later timepoints likely reflects the smaller number of participants at these timepoints, as reflected in the corresponding sample sizes. Data for the induction phase are reported elsewhere.^13^

Depressive symptoms, assessed by MADRS, decreased during induction (Figure 2); improvement was maintained during optimization/maintenance (mean [SD] change from baseline to endpoint of each phase: induction, −12.8 [9.73]; optimization/maintenance, + 0.2 [9.93]). The proportion of responders increased over time during the induction phase, from 15.0% (66/439) on day 8 to 49.2% (224/455) at endpoint. A total of 35.6% (162/455) of participants were in remission at the induction endpoint, and 48.5% (382/788) and 49.6% (551/1110) at week 112 and endpoint of the optimization/maintenance phase, respectively.

Consistent with investigators’ assessments, study participants reported improvement in depressive symptoms based on decrease in PHQ-9 score during induction, with improvement maintained during optimization/maintenance (mean [SD] change from baseline to endpoint of each phase: induction, −5.8 [5.84]; optimization/maintenance, + 0.6 [6.22]) (Figure S17). The percentage of participants who characterized their depressive symptoms as moderately severe to severe, based on PHQ-9 score, decreased from baseline (56.5%, 258/456) to endpoint of the induction phase (20.5%, 93/454) and endpoint of the optimization/maintenance phase (16.2%, 180/1110). The percentage of remitters (PHQ-9 score <5) was 19.8% (90/454) at endpoint of the induction phase and 32.2% (253/785) and 33.9% (376/1110) at week 112 and endpoint, respectively, of the optimization/maintenance phase.

**Figure 3.**
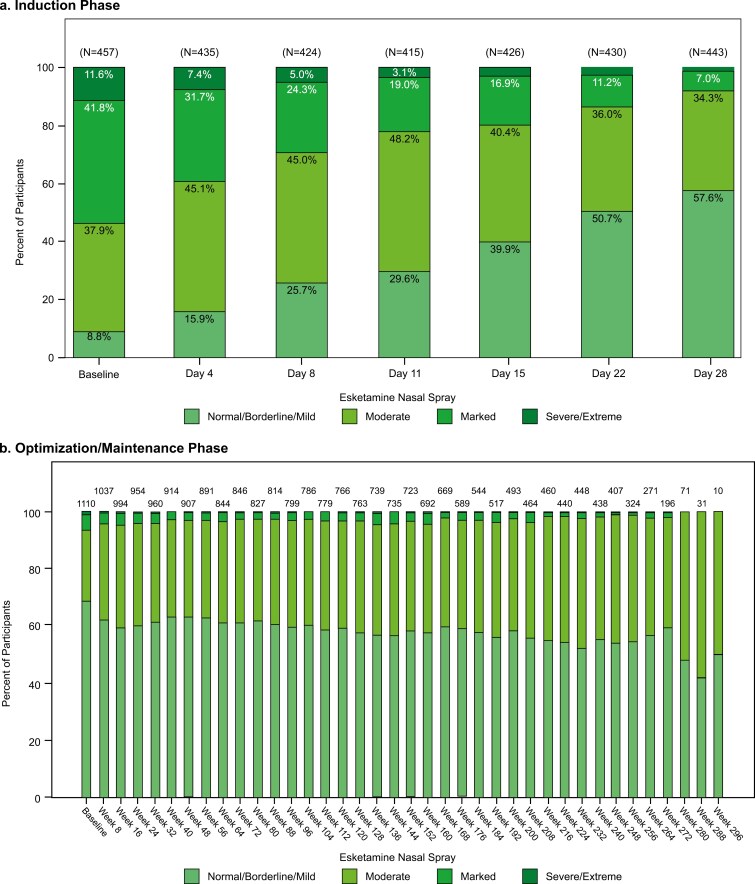
**Clinical Global Impression–Severity (CGI-S): frequency distribution over time**. (**a**) **Induction phase**. (**b) Optimization/maintenance phase**. Notes: Every-8-week data are presented. The visits with fewer than 10 participants are not presented. The CGI-S evaluates the severity of psychopathology on a scale of 0 to 7: 0 = not assessed; 1 = normal (not at all ill); 2 = borderline mentally ill; 3 = mildly ill; 4 = moderately ill; 5 = markedly ill; 6 = severely ill; 7 = among the most extremely ill patients. Data for the induction phase are reported elsewhere.^13^

Illness severity, as assessed by the CGI-S, improved from baseline (median of 5) to induction phase endpoint (median [range] change from baseline, −1.0 [−5; 1]) and was stable over the optimization/maintenance phase (median [range] change from baseline to endpoint: 0.0 [−5; 4]). More than half of the participants had CGI-S scores indicating normal/borderline/mild illness (scores of 1, 2, or 3) at the induction (55.9%) and optimization/maintenance (60.9%) phase endpoints (Figure 3).

#### Functioning and Associated Disability

Functioning and associated disability, assessed by SDS score, improved during induction; improvement was maintained during optimization/maintenance (mean [SD] change from the baseline to endpoint of each phase: induction, −6.4 [7.13]; optimization/maintenance, −0.1 [8.35]) (Figure S18). Based on SDS scores, the percentage of responders was 44.8% (174/388) and 54.7% (593/1084) at induction and optimization/maintenance phase endpoints, respectively, and the percentage of participants in remission was 22.9% (89/388) and 37.0% (401/1084) at the respective phase endpoints.

## DISCUSSION

The long-term safety and efficacy of esketamine nasal spray, combined with an oral antidepressant, was evaluated in the global SUSTAIN-3 study of TRD. The mean duration of intermittent esketamine treatment in this trial was 42.9 months (range up to 79 months). Nearly two-thirds of study participants continued in the SUSTAIN-3 study for 3 years or longer, despite that esketamine could only be administered at in-person clinic visits and the research period overlapped the peak of the COVID-19 pandemic. Importantly, few participants discontinued due to lack of efficacy (5.3% across phases) or an adverse event (6.4% across phases).

The safety profile of esketamine, with continuous intermittent dosing for up to 6.5 years in SUSTAIN-3 (3777 cumulative patient-years), aligns with its established safety profile reported in studies of shorter exposure.^6,12^ Consistent with the safety observations in the 32-week ESCAPE-TRD trial,^25^ most adverse events reported in SUSTAIN-3, including events of dissociation and sedation, were transient, occurring and resolving on a dosing day. Long-term exposure to esketamine yielded no new concerns related to increased BP, renal disorders, lower urinary tract symptoms, or suicidality. Additionally, there was no evidence of impaired cognition, interstitial or ulcerative cystitis, or hepatotoxicity. Safety findings have been confirmed in real-world, naturalistic settings,^26,27^ with no evidence of abuse, misuse, or withdrawal within the context of the current delivery model for esketamine nasal spray.^26^ In surveillance of real-world data, Dart^28^ reported an increasing trend for misuse/abuse of ketamine, but not of esketamine.

Few (2%) participants had serious adverse event(s) related to depression that required hospitalization during this long-term study of esketamine, as also was the case (0.6%) in the ESCAPE-TRD trial.^25^ These data compare favorably to a prior report of patients with TRD receiving standard-of-care treatments, wherein 8% of patients required hospitalization during a much shorter (6-month) period of study.^29^

Incidences of suicide attempts, suicide death, and all-cause mortality are higher among patients with TRD than in the general MDD population.^30,31^ In SUSTAIN-3, 16 patients (1.4%) attempted suicide (0.423 per 100 patient-years), 1 died by suicide (0.0265 per 100 patient-years), and 9 died by any cause (all-cause mortality rate 0.238 per 100 patient-years). For context, the rate of non-fatal suicidal behavior was 4.66 per 100 patient-years and the rate of suicide death was 0.14-0.47 per 100 patient-years in studies assessing several other TRD treatments.^32-35^ The literature-reported rate of all-cause mortality for TRD ranged from 0.79^31^ to 4.6^30^ per 100 patient-years. Of note, the rates of suicide-related behavior and death, and of all-cause mortality observed in SUSTAIN-3 may reflect the effectiveness of maintenance treatment with intermittently-dosed esketamine plus daily antidepressant; the rates may also have been influenced by the entrance criteria, as the parent studies from which participants were recruited had excluded volunteers who reported suicidal ideation with intent or plan within the past 6 months or a history of suicidal behavior within the past year, and by the close clinical follow-up that was provided as a result of participating in this clinical trial.

Cognition remained stable over time for participants <65 years and participants ≥65 years of age, based on performance on most cognitive tests. Of note, decline on measures of higher cognitive function was not observed among participants ≥65 years old. Based on mean change from baseline scores, slowing on simple and choice reaction times occurred among participants aged <65 and ≥65 years. The slowing on reaction time tests increased during optimization/maintenance in both age subgroups and reached magnitudes suggestive of a small effect size by approximately week 200 for participants <65 and at earlier timepoints for participants ≥65. When considering RCI scores, the reaction time performance of many participants fluctuated in and out of “normal” (not clinically meaningful) ranges based on RCI values. The clinical relevance and reliability of the observed slowing on reaction time tests are unclear considering the relatively small sample size and the intraindividual variability in performance, which has been observed as a factor in slowing of processing speed/reaction time in longitudinal studies of older, healthy participants^36^ and of mood disorder patients,^37^ including patients with MDD aged ≥65.^38^ In the absence of a control arm, it cannot be determined whether slowing in reaction time/processing speed is the result of study drug(s), or intrinsic processes among older and/or depressed individuals that contribute to increasing intraindividual variability of performance over time, which in turn result in slowing reaction time/processing speed. The absence of negative effects of esketamine on any aspect of memory, learning, executive function, and working memory in both subgroups makes it unlikely that the subtle changes in simple and choice reaction time reflect the effects of treatment with esketamine.

Measures of depressive symptoms and functioning improved during the first 4 weeks of treatment (induction phase) and were sustained for up to 6.5 years with intermittently-dosed esketamine plus daily antidepressant (during the optimization/maintenance phase). Half of the participants were in (MADRS-defined) remission at the optimization/maintenance phase endpoint. Our findings are consistent with and extend those of SUSTAIN-2, in which a 47% remission rate was observed at 12 months.^12^

The long-term efficacy of esketamine for TRD is further supported by sustained improvements in clinician-assessed severity of illness (CGI) and patient-reported measures of depressive symptoms (PHQ-9) and functionality (SDS). In SUSTAIN-3, the percentage of participants who achieved functional remission (defined as SDS ≤6) was 22.9% (89/388) and 37.0% (401/1084) at the induction and optimization/maintenance phase endpoints, respectively. The latter finding is consistent with that reported in the ESCAPE-TRD trial, in which 37.0% of esketamine-treated participants achieved functional remission at 32 weeks.^39^ In addition, for a study spanning up to 6.5 years in patients with TRD, the proportions of participants who discontinued during optimization/maintenance due to lack of efficacy (4.7%), worsening depression (0.6%), suicide ideation (0.2%), or suicide attempt (0.1%) are relatively low when considered within the context of prior literature reports.

Most participants maintained clinical stability with the 84 mg or 56 mg esketamine dose, administered either weekly or every-other-week. The every-4-week dosing interval was used more frequently over time during the optimization/maintenance phase, from about 10% during the first 6 months to about 20% by the study end. This suggests that some participants may be maintained with longer intervals between doses during long-term treatment. While these figures may be relevant to clinical practice, research study participants typically have more time and access to clinical staff than patients in the community who are treated outside of research settings. These differences should be considered when applying the dosing and scheduling of participants in the trial to community-based clinical care.

Several factors may limit the generalizability of our findings, including the open-label and single-arm design, potential bias of participants electing to proceed from a parent study into this extension study, exclusion of participants with significant psychiatric comorbidities, lack of racial heterogeneity, and level of care participants received (in addition to esketamine), which generally exceeded that in real-world settings. Participants received esketamine and associated clinical care in SUSTAIN-3, with most participants staying in the study; thus, the SUSTAIN-3 results cannot determine the optimal duration of treatment in clinical practice. The study design did not allow extending the dosing interval beyond 4 weeks, as may be observed in real-world practice. Additional analyses are warranted to further assess disease characteristics and their course over time, the impact of comorbidities, and the optimal dosing regimen.

In summary, no new safety signals were identified during long-term treatment with intermittently-dosed esketamine, combined with daily antidepressant, and improvement in depression generally persisted among participants who remained on maintenance treatment. Taken together, these data from SUSTAIN-3 add to the evidence supporting the safety of long-term use of intermittently-dosed esketamine for treating TRD.

## Supplementary Material

pyaf027_suppl_Supplementary_Material

## Data Availability

The data sharing policy of Johnson & Johnson is available at https://innovativemedicine.jnj.com/our-innovation/clinical-trials/transparency. As noted on this site, requests for access to the study data can be submitted through Yale Open Data Access [YODA] Project site at http://yoda.yale.edu.
