# Supplementary material for: Safety and efficacy with esketamine in treatment-resistant depression: long-term extension study
Source: Int J Neuropsychopharmacol. 2025 May 4;28(6):pyaf027. doi: 10.1093/ijnp/pyaf027 (PMC12143125; doi:10.1093/ijnp/pyaf027)
Supplement: pyaf027_suppl_Supplementary_Material [file pyaf027_suppl_supplementary_material.pdf]

## Supplementary Materials

Zaki N, Chen L, Rosanne Lane R, et al. Safety and efficacy with esketamine in treatment-resistant depression: long-term extension study. *International Journal of Neuropsychopharmacology*. Published online May 4, 2025. doi: 10.1093/ijnp/pyaf027.


| | |
|---|---|
| Methods S1. | List of Institutional Review Boards and Independent Ethics Committees |
| Figure S1. | SUSTAIN-3 Study Design |
| Table S1. | Study Drug Dosing During SUSTAIN-3 |
| Methods S2. | Description of Safety Measures and Efficacy Scales |
| Table S2. | Number of Participants Who Enrolled from Previous Studies by Entry Point (SUSTAIN-3 Study: All Enrolled Analysis Set) |
| Figure S2. | Frequency Distribution of Participants Exposure to Esketamine Nasal Spray During Induction and Optimization/Maintenance Phases |
| Figure S3. | Dosing Frequency of Esketamine Nasal Spray Over Time |
| Table S3. | Concomitant Oral Antidepressants Used in SUSTAIN-3 |
| Table S4. | Treatment-Emergent Serious Adverse Events During the Induction and Optimization/Maintenance Phases |
| Results S1. | Deaths |
| Table S5. | Treatment-Emergent Adverse Events Leading to Discontinuation of Esketamine During the Induction and Optimization/Maintenance Phases |
| Figure S4. | Event Rate of Treatment-Emergent Adverse Events of Dissociation Persisting Beyond 2 Hours |
| Figure S5. | Event Rate of Treatment-Emergent Hepatic Adverse Events |
| Figure S6. | Incidence of Increased Blood Pressure Adverse Events Over Time |
| Results S2. | Results of Cognitive Assessments |

Figure S7. Detection – Attention (Simple Reaction Time) by Age Group Over the Induction and Optimization/Maintenance Phases of SUSTAIN-3

Figure S8. Identification – Attention (Choice Reaction Time) by Age Group Over the Induction and Optimization/Maintenance Phases of SUSTAIN-3

Figure S9. One Card Learning– Visual Learning by Age Group Over the Induction and Optimization/Maintenance Phases of SUSTAIN-3

Figure S10. One Back Test– Working Memory by Age Group Over the Induction and Optimization/Maintenance Phases of SUSTAIN-3

Figure S11. Groton Maze Learning Test – Executive Function by Age Group Over the Induction and Optimization/Maintenance Phases of SUSTAIN-3

Figure S12. HVLT-R Total Recall by Age Group Over the Induction and Optimization/Maintenance Phases of SUSTAIN-3

Figure S13. HVLT-R Delayed Recall by Age Group Over the Induction and Optimization/Maintenance Phases of SUSTAIN-3

Figure S14. HVLT-R Total Number of True Positives by Age Group Over the Induction and Optimization/Maintenance Phases of SUSTAIN-3

Figure S15. HVLT-R Recognition Discrimination Index by Age Group Over the Induction and Optimization/Maintenance Phases of SUSTAIN-3

Table S6. Effect Size (Cohen's d) for Cogstate Tests Scored on Basis of Processing Speed by Age Subgroup

Table S7. RCI Scores for Detection and Identification During Optimization/Maintenance by Age Subgroup (Number of Participants)

Figure S16. Incidence of Columbia-Suicide Severity Rating Scale (C-SSRS) Scores Over Time

Figure S17. Mean (± SE) Patient Health Questionnaire 9-Item (PHQ-9) Total Score (Observed Cases)

Figure S18. Mean (± SE) Sheehan Disability Scale Total Score (Observed Cases)


**This supplementary material has been provided by the authors to give readers additional information about their work.**

**Methods S1. List of Institutional Review Boards and Independent Ethics Committees**

**ARGENTINA**

CEI Fundación Rusculleda
Comité de Etica CAICI-CIAP
Comité de Ética de CER Investigaciones Clínicas (CECIC)
Comite de Etica del Instituto Medico Platense (CEDIMP)
Comité de Etica en Investigación (CEI-INAPSI)
Comité de Etica en Investigacion Burzaco
Comité de Etica en Investigación Clínica Privada de Salud Mental Stanta Teresa de Avila
Comité Independiente de Ética para Ensayos en Farmacología Clínica Fundación de Estudios Farmacológicos y de Medicamentos
Comité Institucional de Ética en Investigación en Salud-CIEIS Hospital Italiano

**AUSTRALIA**

Alfred Health Human Ethics Committee
Bellberry Limited
Northern Adelaide Local Health Network - IRB/IEC

**AUSTRIA**

der Stadt Wien gemäß KAG, AMG und MPG
Med.Universität Wien

**BELGIUM**

AZ Sint-Lucas IRB
AZ St.-Jan Brugge
Comite d'Ethique Cliniques Universitaires De Mont Godinne
Comité d'Ethique Hospitalo-Facultaire
Ethisch Comité OLV Ziekenhuis Aalst
Sint-Franciskusziekenhuis IRB
UZ Brussel IRB

**BRAZIL**

CEP do Hospital de Clinicas de Porto Alegre – HCPA / UFRGS
Comite de Etica em Pesquisa - Instituto de Psiquiatria da Universidade Federal do Rio de Janeiro
Comitê de Ética em Pesquisa da Faculdade de Medicina do ABC
Comite de Etica em Pesquisa da UNIFESP/EPM
Comite de Etica em Pesquisa do Complexo Hospitar HUOC/PROCAPE
Comite de Etica em Pesquisa do Hospital Universitario Sao Jose - Faculdade de Ciencias Medicas de Minas Gerais
Comite de Etica em Pesquisa do Instiruto de Neurologia de Curitiba
Comitê de Ética em Pesquisa do Investiga - Instituto de Pesquisas

**BRAZIL (continued)**

Comite de Etica em Pesquisa do Hospital Universitario Walter Cantidio
Comite de Etica em Pesquisa em Seres Humanos da Universidade de Passo Fundo
Comite de Etica em Pesquisas do Hospital Pro-Cardiaco
National Committee of Ethics in Research (CONEP)

**BULGARIA**

Ethics Committee for Clinical Trials, Sofia

**CANADA**

CAMH Research Ethics Board
Queen's University, Health Sciences and Affiliated Hospitals Research Ethics Board
The Royal’s Institute of Mental Health Research Ethics Board
UBC Clinical Research Ethics Board (CREB)

**CZECH REPUBLIC**

Eticka komise nestatniho zdravotnickeho zarizeni Research Site s.r.o
Etická komise NZZ Clintrial, s.r.o.
Eticka komise pro multicentricke klinicke hodnoceni Fakultni nemocnice v Motole

**ESTONIA**

Research Ethics Committee of the National Institute for Health Development

**FINLAND**

Naisten Lasten ja Psykiatrian Eettinen Toimikunta

**FRANCE**

CPP ile de France VIII

**GERMANY**

Ethikkommission bei der Bayerischen Landesärztekammer
Ethik-Kommission bei der Landesärztekammer Rheinland-Pfalz
Ethik-Kommission der Ärztekammer Westfalen-Lippe und der Medizinischen Fakultät der Westfälischen Wilhelms-Universität Münster
Ethik-Kommission der Landesärztekammer Brandenburg
Ethikkommission der Sächsischen Landesärztekammer
Landesamt für Gesundheit und Soziales Berlin Geschäftsstelle der Ethik-Kommission des Landes Berlin

**HUNGARY,**

Medical Research Council Ethics Committee for Clinical Pharmacology

**ITALY**

Comitato Etico dell'Università "La Sapienza" Sezione Azienda Ospedaliera Sant'Andrea
Comitato Etico Ospedale San Raffaele
Comitato Etico per la sperimentazione clinica della Provincia di Vicenza (CESC-VI)
Comitato Etico Regione Liguria
Comitato Etico Regione Toscana - Area Vasta Sud-Est (C.E.A.V.S.E.)

**LITHUANIA**

Lithuanian Bioethics Committee

**MALAYSIA**

Medical Research and Ethics Committee (MREC)
Medical Research Ethics Committee, University Malaya Medical Centre

**MEXICO**

Comité de Etica en Investigación de Hospitales Mac-Bernardette
Comité de Ética en Investigación de la Facultad de Medicina y Hospital Universitario de la Universidad Autónoma de Nuevo León
Comité de Ética en Investigación del Hospital Aranda de la Parra S.A. de C.V.
Comité de Etica en Investigación del Hospital La Mision SA de CV
Comité de Etica en Investigación del Mexico Centre for Clinical Research SA de CV
Comité de Ética en Investigación Hospital Ignacio Morones Prieto
Comité de la Clínica Bajío CLINBA, S.C.

**POLAND**

Komisja Bioetyczna przy OIL w Warszawie
Komisja Bioetyczna przy Okregowej Izbie Lekarskiej w Gdansku

**REPUBLIC OF KOREA**

Chonnam National University Hospital Institutional Review Board
Korea University Anam Hospital IRB
Kyung Hee University Medical Center IRB
Samsung Medical Center IRB

**SLOVAKIA**

Eticka komisia Bratislavskeho samospravneho kraja
Eticka komisia Liptovskej nemocnice s poliklinikou MUDr. Ivana Stodolu
Eticka komisia Nemocnice s poliklinikou sv. Barbory Roznava
Eticka komisia Presovskeho samospravneho kraja
Nezavisla eticka komisia Banskobystrickeho samospravneho kraja

**SOUTH AFRICA**

Medicine Control Council, Pretoria
Pharma-Ethics, Pretoria
University of Pretoria, Faculty of Health Sciences

**SPAIN**

Comité Ético de Investigación Clínica del Hospital Vall d'Hebrón

**SWEDEN**

Regionala Etikprövningsnämnden i Lund

**TAIWAN**

Chang Gung Medical Foundation Institutional Review Board
Institutional Review Board, Chung Shan Medical University Hospital
Institutional Review Board, Taipei Veterans General Hospital
Mackay Memorial Hospital Institutional Review Board
Taipei Medical University Joint Institutional Review Board

**TURKEY**

Canakkale Onsekiz Mart University Clinical Research Ethics Committee
Şişli Hamidiye Etfal Eğitim ve Araştırma Hastanesi Klinik Araştırmalar Etik Kurulu

**UNITED KINGDOM**

Ashgate Medical Practice Ethics Committee
Derbyshire Healthcare NHS Foundation Trust
Health Research Authority IRB/EC
Oxford Health NHS Foundation Trust
South Central- Oxford C
South London and Maudsley NHS Foundation Trust

**UNITED STATES**

Baylor College of Medicine IRB, Houston, TX
Butler Hospital IRB, Providence, RI
Creighton University Institutional Review Board, Omaha, NE
Hartford Hospital IRB, Hartford, CT
Human Research Protection Program, New Haven, CT
Johns Hopkins Medicine Institutional Review Board, Baltimore, MD
KU Human Subjects Committee, Wichita, KS
Loyola University Chicago Health Sciences Division, Protection of Human Subjects, Chicago, IL
Office of Regulatory Affairs, University of PA
Office of Research Integrity, Charleston, SC
Sharp HealthCare IRB, San Diego, CA
Sterling Institutional Review Board
Sterling IRB, Atlanta, GA

**UNITED STATES (continued)**

University of Iowa IRB
University of Virginia Institutional Review Board for Health Science Research
UT Southwestern IRB, Dallas, TX
Western Institutional Review Board, Puyallup, WA

**Figure S1. SUSTAIN-3 Study Design**

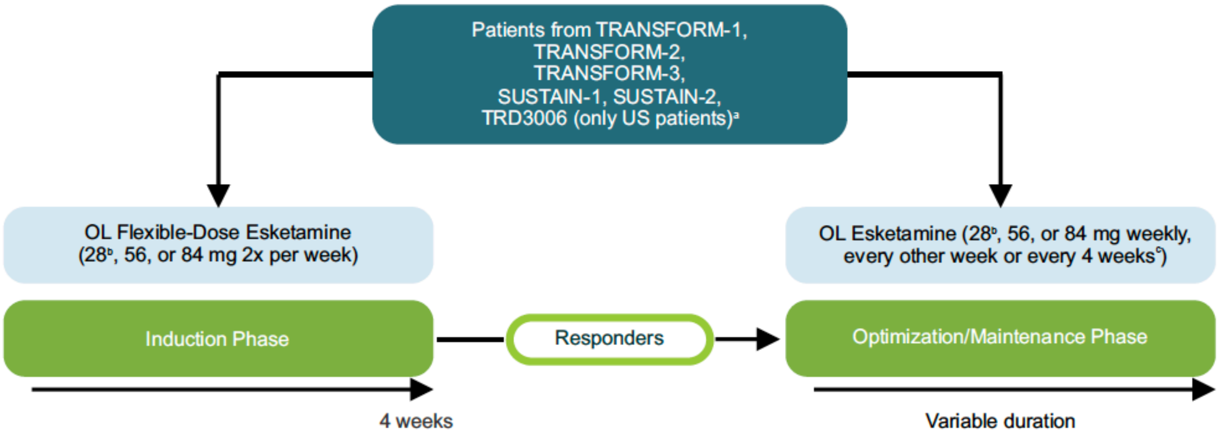


OL = open label.

a. clinical trials.gov identifiers (study name): NCT02417064 (TRANSFORM-1), NCT02418585 (TRANSFORM-2), NCT02422186 (TRANSFORM-3), NCT02493868 (SUSTAIN-1), NCT02497287 (SUSTAIN-2), NCT03434041 (TRD3006)

b. 28 mg dose only an option for participants ≥65 years

c. Based on clinical global impression - severity (CGI-S) and tolerability

Notes: Participants were eligible to enroll into the Induction Phase or the Optimization/Maintenance Phase of SUSTAIN-3 based on their status at the end of the parent study.

Based on the parent study the participant entered SUSTAIN-3:

- From TRANSFORM-1 or TRANSFORM-2 study:
  - Participant had completed the induction phase and the 2-week follow-up phase visit; or
  - Participant completed the induction phase and was a responder, and study SUSTAIN-1 was terminated.
- From SUSTAIN-1 study:
  - Participant relapsed during the maintenance phase; or
  - Participant was in the induction phase of the SUSTAIN-1 study when the study was terminated and, after completion of the induction phase, was determined to be a responder; or
  - Participant was in the optimization or maintenance phases at the time the study was terminated; or
  - At week 16 of optimization, the participant was not eligible to proceed to the maintenance phase and sponsor had approved participant's entry into SUSTAIN-3; or
  - Participant was in the induction phase and after completion of induction phase was determined to not meet response criteria, and sponsor had approved participant's entry into SUSTAIN-3.
- From SUSTAIN-2 study:
  - Participant completed SUSTAIN-2 study optimization/maintenance phase; or
  - Participant was in the induction phase of the SUSTAIN-2 study when the study was terminated and, after completion of the induction phase, was determined to be a responder; or
  - Participant was in the optimization/maintenance phase at the time the study was terminated; or
  - Participant was in the induction phase and did not meet criteria for response, and sponsor had approved participant's entry into SUSTAIN-3.
- From TRANSFORM-3 study: Participant was in the induction phase of the TRANSFORM-3 study at the time enrollment into the SUSTAIN-2 study was closed and, after completion of the induction phase, was determined to be a responder or did not meet the criteria for response.
- From TRD3006 study (US Study sites only):
  - Participant completed the induction phase and was a responder; or
  - Participant completed the induction phase and did not meet the response criteria and sponsor had approved participant's entry into SUSTAIN-3.

## Table S1. Study Drug Dosing During SUSTAIN-3

### Induction Phase

All eligible participants self-administered the intranasal study drug twice a week for 4 weeks at treatment sessions at the study site. Treatment sessions should not have taken place on consecutive days.

Esketamine dose titration in the Induction Phase for participants <65years of age is described in Table 1:

**Table 1. Induction Phase Dose Titration of Esketamine Nasal Spray – Participants < 65 Years**

| Day | Dose | Dose Titration Guidance |
|---|---|---|
| Day 1 | 56 mg | |
| Day 4 | 56 or 84 mg | The dose may remain at 56 mg or be increased to 84 mg, as determined by the investigator based on efficacy and tolerability. |
| Day 8, 11, 15, 18, 22, 25 | 56 or 84 mg | The dose may be increased to 84 mg (if previous dose was 56 mg), remain the same, or be reduced to 56 mg (if previous dose was 84 mg), as determined by the investigator based on efficacy and tolerability |

Esketamine dose titration in the Induction Phase for participants ≥65years of age is described in Table 2:

**Table 2. Induction Phase Dose Titration of Esketamine Nasal Spray – Participants ≥ 65 Years**

| Day | Dose | Dose Titration Guidance |
|---|---|---|
| Day 1 | 28 mg | |
| Day 4 | 28 or 56 mg | The dose may remain at 28 mg or be increased to 56 mg, as determined by the investigator based on efficacy and tolerability. |
| Day 8, 11, 15, 18, 22, 25 | 28, 56, or 84 mg | The dose may remain the same, or be increased or reduced by 28 mg from the previous dosing session, as determined by the investigator based on efficacy and tolerability |

## Optimization/Maintenance Phase

Participants self-administered the intranasal study drug at treatment sessions at the study site. Treatment sessions should not have taken place on consecutive days.

For the first 4 weeks of the optimization/maintenance phase (week 1 to week 4):

- Participants from the induction phase of SUSTAIN-3, who entered the optimization/maintenance phase, continued on the same dose of esketamine from the induction phase and had a weekly treatment session frequency (i.e., reduced frequency from the twice-weekly frequency in the induction phase).
- Participants who were responders at the end of the induction phase of TRANSFORM-1, TRANSFORM-2, or Study TRD3006 (clinical trials.gov: NCT03434041; US sites only) who entered the optimization/maintenance phase had a weekly treatment session frequency (i.e., reduced frequency from the twice-weekly frequency in the induction phase). However, as the study drug in these 3 parent studies was blinded at the time of entry into SUSTAIN-3, participants who entered the optimization/maintenance phase were to start at 56 mg. The dose remained at 56 mg or was increased to 84 mg, as determined by the investigator based on efficacy and tolerability.
- With sponsor approval, participants from the SUSTAIN-1 and SUSTAIN-2 studies who entered directly from the induction phase and who did not meet the criteria for response in those studies may have entered the optimization/maintenance phase of SUSTAIN-3 and had a weekly treatment session frequency from week 1 to week 4 (i.e., reduced frequency from the twice-weekly frequency in the induction phase). In addition, a one-time dose change was allowed at study entry.
- With sponsor approval, participants from Study TRD3006 (US sites only) who entered directly from induction phase and did not meet criteria for response in that study may have entered the optimization/maintenance phase. However, as the Study TRD3006 study drug was blinded at the time of entry into the current study, participants who entered the optimization/maintenance phase from Study TRD3006 started at 56 mg. The dose remained at 56 mg or was increased to 84 mg, as determined by the investigator based on efficacy and tolerability. Participants had a weekly treatment session frequency from week 1 to week 4 (i.e., reduced frequency from the twice-weekly frequency in the induction phase).
- Participants who entered the optimization/maintenance phase from the TRANSFORM-3 study also had a weekly treatment session frequency. However, as the TRANSFORM-3 study drug was blinded at the time of entry into SUSTAIN-3, the dose of esketamine was administered as outlined in Table 3.

**Table 3. Optimization/Maintenance Phase Week 1 to 4: Dose Titration of Esketamine for Responder Participants Entering from TRANSFORM-3**

| Week | Dose | Dose Titration Guidance |
|---|---|---|
| Week 1 | 28 mg | |
| Week 2 | 28 or 56 mg | The dose may remain at 28 mg or be increased to 56 mg, as determined by the investigator based on efficacy and tolerability |
| Week 3 and 4 | 28, 56 or 84 mg | The dose may remain the same or be increased or reduced by 28 mg from the previous dosing session, as determined by the investigator based on efficacy and tolerability. For those who have had a prior down titration from a higher dose, a dose increase by 28 mg is allowed based on clinical judgment. |

- Participants who entered the optimization/maintenance phase from SUSTAIN-1 (Direct Entry) or SUSTAIN-2 who were ongoing in the optimization, maintenance, or optimization/maintenance phase, respectively, had the option to have their current dosing frequency adjusted at the time of entry into SUSTAIN-3 and remained on the selected frequency from week 1 to week 4 (inclusive). A one-time dose change was permitted at study entry.
- Participants who entered the optimization/maintenance phase from SUSTAIN-1 (Transferred Entry) started at 56 mg. The dose remained at 56 mg or was increased to 84 mg, as determined by the investigator based on efficacy and tolerability. In addition, participants had the option to have their current dosing frequency adjusted at the time of entry into SUSTAIN-3 and remained on the selected frequency from week 1 to week 4 (inclusive).

After week 4 (i.e., starting at week 5), based on the investigator's clinical judgment, the dose of esketamine for all participants could have been adjusted based upon efficacy and tolerability.

Starting at week 4, the frequency for subsequent treatment sessions was adjusted (if applicable) based on the algorithm outlined in Table 4 at fixed, 2-week intervals.

**Table 4. Algorithm for Adjusting Treatment Session Frequency (if applicable) Starting at Week 4**

| Current treatment session frequency | CGI-S score at current visit[a] | |
|---|---|---|
| | ≤3 | >3 |
| Weekly | Change to every other week frequency | No change in frequency |
| Every other week | No change in frequency or change to every 4 weeks per clinical judgment | Change to weekly frequency |
| Every 4 weeks | No change in frequency | Change to weekly or every other week frequency per clinical judgment |

[a] Note: Although the CGI-S was administered every 2 weeks from week 4 through the end of the Optimization/Maintenance Phase, adjustment of the treatment session frequency was only permitted at the fixed, 2-week interval (based on CGI-S performed at that visit) and every 4 weeks for participants dosed at the 4-week interval. For example, if at week 4 a participant was currently at a weekly treatment session frequency and the CGI-S score at week 4 was a 2, the treatment session frequency was changed from weekly to every other week (i.e., the next treatment session for this participant was at week 6).

## Methods S2. Description of Safety Measures and Efficacy Scales

### Safety Measures

The Columbia Suicide Severity Rating Scale (C-SSRS) was used to assess potential suicidal ideation and behavior. The C-SSRS is a measure of the spectrum of suicidal ideation and behavior that was developed in the National Institute of Mental Health Treatment of Adolescent Suicide Attempters Study to assess severity and track suicidal events through any treatment [Posner et al., 2007] It is a clinical interview providing a summary of both suicidal ideation and behavior that can be administered during any evaluation or risk assessment to identify the level and type of suicidality present. The C-SSRS can also be used during treatment to monitor for clinical worsening. In this study, the C-SSRS assessments (induction: all visits; optimization/maintenance: days 1, 8, 15, 22 then every-2-weeks, and at early withdrawal/study end) used the Since Last Visit version, which assesses suicidal ideation and behavior since the participant's last visit.

Modified Observer's Assessment of Alertness/Sedation (MOAA/S) was used to measure treatment-emergent sedation, with correlation to levels of sedation defined by the American Society of Anesthesiologists (ASA) continuum. The MOAA/S scores range from 0=no response to painful stimulus (corresponds to ASA continuum for general anesthesia) to 5=readily responds to name spoken in normal tone (awake; corresponds to ASA continuum for minimal sedation). The MOAA/S, was performed every 15 minutes from predose to 1-hour postdose (induction: each dosing day; optimization/maintenance: days 1,8,15, and 22).

Cognition was assessed (induction: baseline and day 28; optimization/maintenance: day 15, every 12-weeks, and at early withdrawal/study end) using the Cogstate computerized test battery and the Hopkins Verbal Learning Test-Revised (HVLT-R). The computerized cognitive battery provides assessment of multiple cognitive domains, including attention, visual learning and memory, and executive function. The tests use culture-neutral stimuli, enabling use in multilingual/multicultural settings. The computerized battery includes:

- Simple and choice reaction time tests; scored for speed of response (mean of the log 10-transformed reaction times for correct responses)
- Visual episodic memory; visual recall test scored using arcsine transformation of the proportion of correct responses
- Working memory (n back); scored for speed of correct response (mean of the log 10-transformed reaction times for correct responses)
- Executive function; maze/sequencing test, scored for total number of errors

All measures have been validated against traditional neuropsychological tests and are sensitive to the effects of various drugs on cognitive performance, including alcohol and benzodiazepines. Completing the cognitive battery requires approximately 25 minutes.

The HVLT-R, a measure of verbal learning and memory, is a 12-item word list recall test. Administration includes 3 learning trials, a delayed recall (20-minute) trial, and a 24-word recognition list (including 12 target and 12 foil words) [Benedict et al., 1998] The test administrator reads instructions and word lists aloud, and records words recalled/recognized by the participant. Scores include learning, delayed recall, and recognition. The HVLT-R is a well-validated and widely used measure of verbal episodic memory. The tests were administered in the following order: HVLT-R, computerized cognitive test battery, and HVLT-R Delayed.

**Efficacy Scales**

The Montgomery-Åsberg Depression Rating Scale (MADRS) is a clinician-rated scale designed to measure depression severity and detects changes due to antidepressant treatment [Montgomery & Åsberg, 1979]. The MADRS scale consists of 10 items, each of which is scored from 0 (item not present or normal) to 6 (severe or continuous presence of the symptoms), for a total possible score of 60. Higher scores represent a more severe condition. The MADRS evaluates apparent sadness, reported sadness, inner tension, sleep, appetite, concentration, lassitude, inability to feel (interest level), pessimistic thoughts, and suicidal thoughts. The test

exhibits high inter-rater reliability. The structured interview guide for the MADRS (SIGMA) was used for each administration.

The Clinical Global Impression – Severity (CGI-S) provides an overall clinician-determined summary measure of the severity of the participant's illness that takes into account all available information, including knowledge of the participant's history, psychosocial circumstances, symptoms, behavior, and the impact of the symptoms on the participant's ability to function [Guy 1976]. The CGI-S evaluates the severity of psychopathology on a scale of 0 to 7. Considering total clinical experience, a participant is assessed on severity of mental illness at the time of rating according to: 1=normal (not at all ill); 2=borderline mentally ill; 3=mildly ill; 4=moderately ill; 5=markedly ill; 6=severely ill; 7=among the most extremely ill patients. The CGI-S permits a global evaluation of the participant's condition at a given time.

The Patient Health Questionnaire 9-item (PHQ-9) is a 9-item, patient-reported outcome measure to assess depressive symptoms [Spitzer et al., 1999]. The scale scores each of the 9 symptom domains of the DSM-5 MDD criteria and it has been used both as a screening tool and a measure of response to treatment for depression. Each item is rated on a 4-point scale (0=not at all, 1=several days, 2=more than half the days, and 3=nearly every day). The participant's item responses are summed to provide a total score (range of 0 to 27), with higher scores indicating greater severity of depressive symptoms.

The Sheehan Disability Scale (SDS) was used to assess the secondary objective of functional impact and associated disability. The SDS, a patient-reported outcome measure, is a 5-item questionnaire which has been widely used and accepted for assessment of functional impairment and associated disability Leon et al., 1997]. The first three items assess disruption of (1) work/school, (2) social life, and (3) family life/home responsibilities using a 0-10 rating scale. The score for the first three items is summed to create a total score of 0-30, where a higher score indicates greater impairment. It also has one item on days lost from school or work and one item on days when underproductive.

***References***

Benedict RHB, Schretlen D, Groninger L, Brandt J. Hopkins Verbal Learning Test - Revised: normative data and analysis of inter-form and test-retest reliability. Clin Neuropsychol. 1998;12:43-55.

Guy W. ECDEU Assessment Manual for Psychopharmacology. US Department of Health, Education, and Welfare: Rockville, MD; 1976.

Leon AC, Olfson M, Portera L, Farber L, Sheehan DV. Assessing psychiatric impairment in primary care with the Sheehan Disability Scale. Int J Psychiatry Med. 1997;27(2):93-105. doi: 10.2190/T8EM-C8YH-373N-1UWD

Montgomery SA, Äsberg M. A new depression scale designed to be sensitive to change. Br J Psychiatry. 1979;134:382-389. doi: 10.1192/bjp.134.4.382.

Posner K, Oquendo MA, Gould M, Stanley B, Davies M. Columbia Classification Algorithm of Suicide Assessment (C-CASA): classification of suicidal events in the FDA's pediatric suicidal risk analysis of antidepressants. Am J Psychiatry. 2007;164:1035-1043.

Spitzer RL, Kroenke K, Williams JB. Validation and utility of a self-report version of PRIME-MD: the PHQ primary care study. Primary Care Evaluation of Mental Disorders. Patient Health Questionnaire. JAMA. 1999;282(18):1737-1744. doi: 10.1001/jama.282.18.1737.

**Table S2. Number of Participants Who Enrolled from Previous Studies by Entry Point (SUSTAIN-3 Study: All Enrolled Analysis Set)**

| Parent Study | Esketamine Nasal Spray N=1148 |
|---|---|
| TRANSFORM-1 | 132 (11.5%) |
| Entry at induction phase | 132 (11.5%) |
| Entry at optimization/maintenance phase | 0 |
| | |
| TRANSFORM-2 | 51 (4.4%) |
| Entry at induction phase | 51 (4.4%) |
| Entry at optimization/maintenance phase | 0 |
| | |
| TRANSFORM-3 | 4 (0.3%) |
| Entry at induction phase | 3 (0.3%) |
| Entry at optimization/maintenance phase | 1 (0.1%) |
| | |
| SUSTAIN-1 | 469 (40.9%) |
| Entry at induction phase | 245 (21.3%) |
| Entry at optimization/maintenance phase | 224 (19.5%) |
| | |
| SUSTAIN-2 | 467 (40.7%) |
| Entry at induction phase | 12 (1.0%) |
| Entry at optimization/maintenance phase | 455 (39.6%) |
| | |
| TRD3006 | 25 (2.2%) |
| Entry at induction phase | 15 (1.3%) |
| Entry at optimization/maintenance phase | 10 (0.9%) |

ClinicalTrials.gov identifiers: NCT02417064, NCT02418585, NCT02422186, NCT02493868, NCT02497287, NCT03434041

**Figure S2. Frequency Distribution of Participants Exposure to Esketamine Nasal Spray During Induction and Optimization/Maintenance Phases**

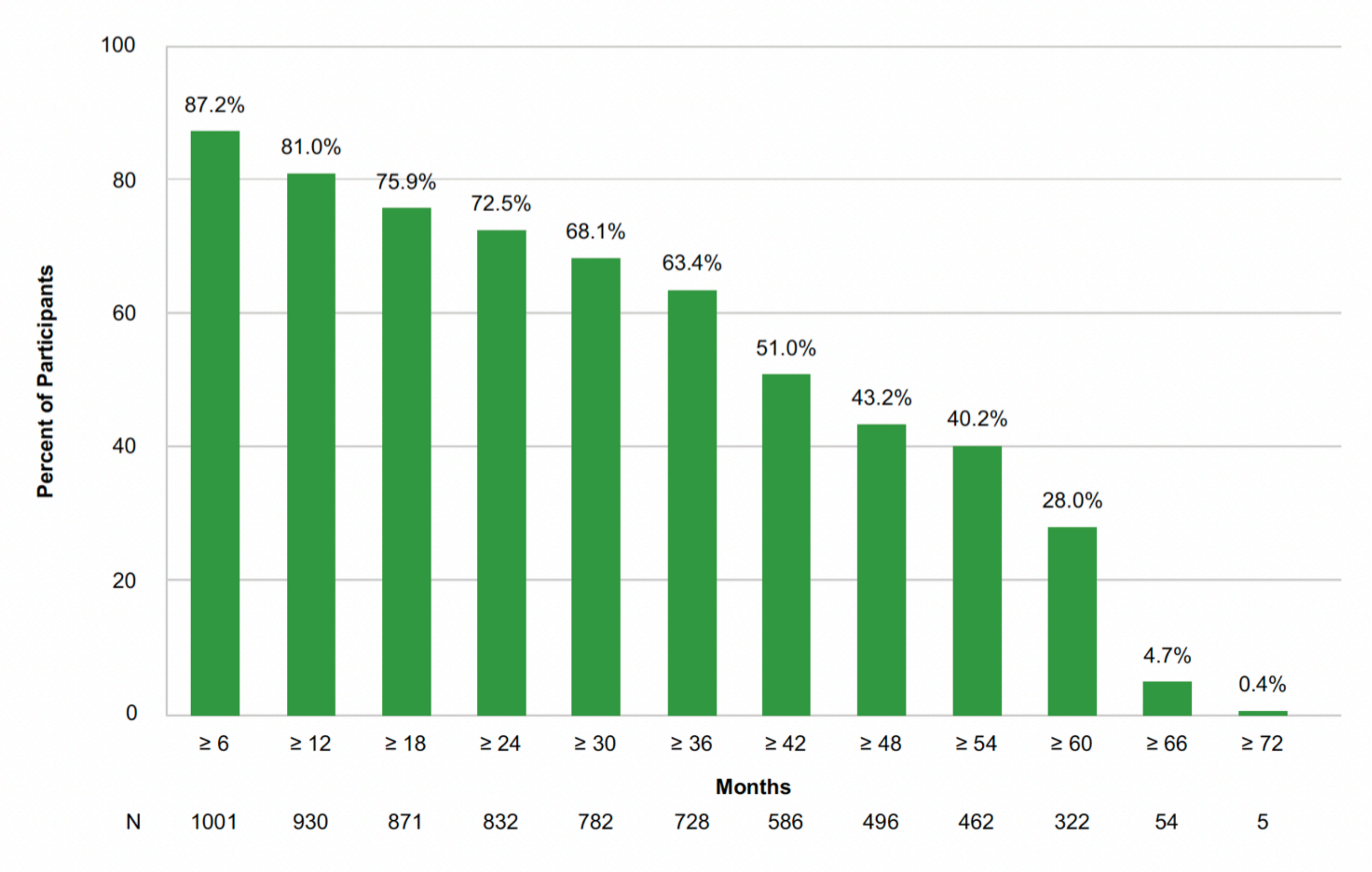

**Figure S3.** **Dosing Frequency of Esketamine Nasal Spray Over Time**

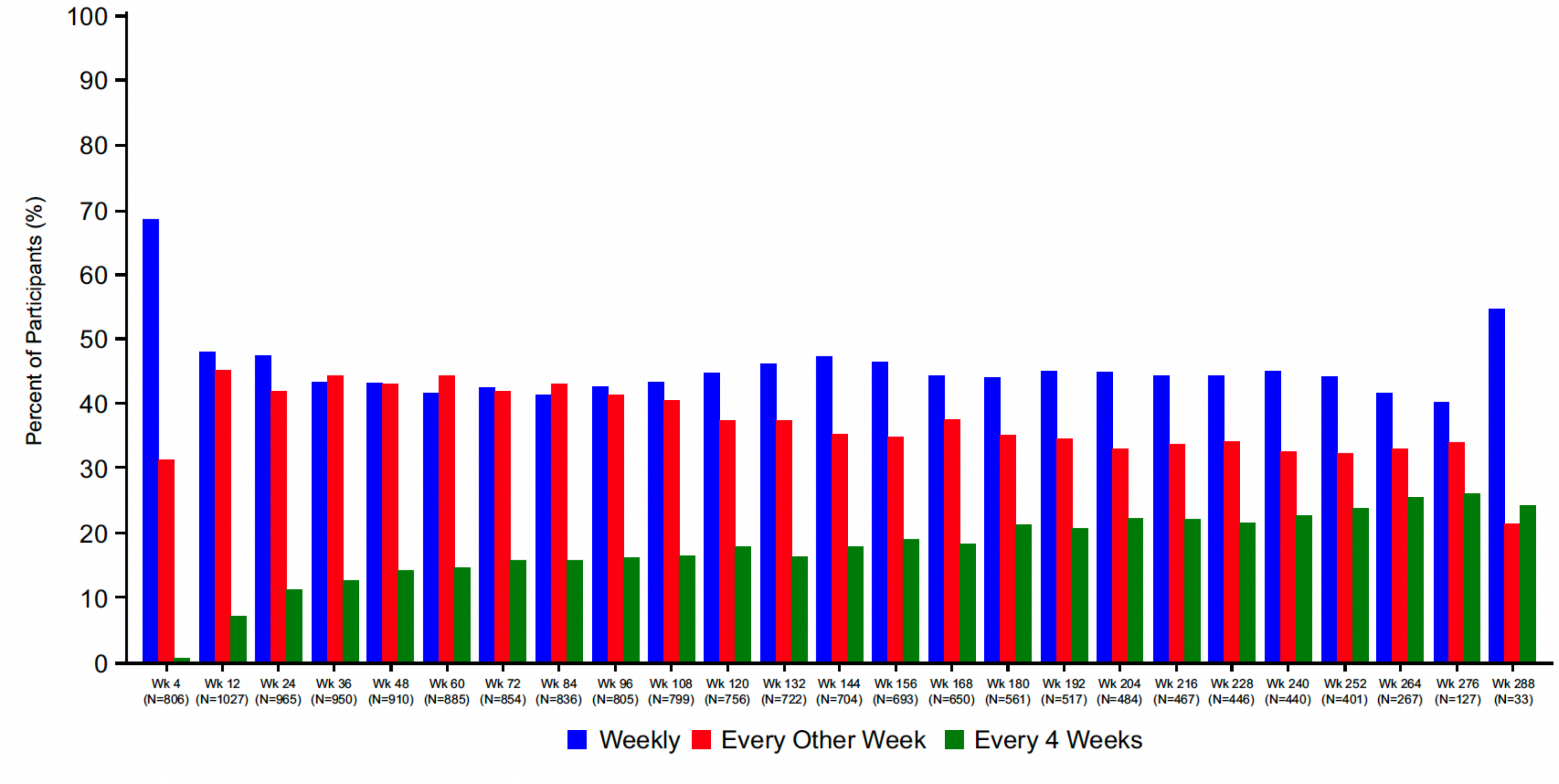


Note: Figure presents the data at specific visits. The visits with fewer than 10 participants are not presented.

**Table S3. Concomitant Oral Antidepressants Used in SUSTAIN-3**

| | |
|---|---|
| **Induction Phase (N = 458)** | |
| Duloxetine | 157 (34.3%) |
| Venlafaxine | 86 (18.8%) |
| Escitalopram | 79 (17.2%) |
| Sertraline | 79 (17.2%) |
| Trazodone | 27 (5.9%) |
| Bupropion | 22 (4.8%) |
| Vortioxetine | 20 (4.4%) |
| Mirtazapine | 14 (3.1%) |
| Fluoxetine | 11 (2.4%) |
| Paroxetine | 7 (1.5%) |
| Citalopram | 6 (1.3%) |
| Amitriptyline | 4 (0.9%) |
| Nortriptyline | 4 (0.9%) |
| Vilazodone | 4 (0.9%) |
| Desvenlafaxine | 3 (0.7%) |
| Fluvoxamine | 3 (0.7%) |
| Levomilnacipran | 3 (0.7%) |
| Clomipramine | 2 (0.4%) |
| Imipramine | 2 (0.4%) |
| Moclobemide | 1 (0.2%) |
| Nefazodone | 1 (0.2%) |
| Opipramol | 1 (0.2%) |
| Tianeptine | 1 (0.2%) |
| **Optimization/Maintenance Phase (N = 1110)** | |
| Duloxetine | 407 (36.7%) |
| Venlafaxine | 307 (27.7%) |
| Escitalopram | 291 (26.2%) |
| Sertraline | 206 (18.6%) |
| Bupropion | 124 (11.2%) |
| Trazodone | 92 (8.3%) |
| Vortioxetine | 78 (7.0%) |
| Mirtazapine | 73 (6.6%) |
| Fluoxetine | 45 (4.1%) |
| Amitriptyline | 29 (2.6%) |
| Agomelatine | 25 (2.3%) |
| Desvenlafaxine | 25 (2.3%) |
| Paroxetine | 24 (2.2%) |
| Citalopram | 17 (1.5%) |
| Nortriptyline | 16 (1.4%) |
| Vilazodone | 16 (1.4%) |
| Clomipramine | 13 (1.2%) |
| Fluvoxamine | 10 (0.9%) |

| | |
|---|---|
| Levomilnacipran | 10 (0.9%) |
| Imipramine | 6 (0.5%) |
| Doxepin | 5 (0.1%) |
| Tranylcypromine | 4 (0.4%) |
| Dosulepin | 3 (0.3%) |
| Desipramine | 2 (0.2%) |
| Mianserin | 2 (0.2%) |
| Nefazodone | 2 (0.2%) |
| Reboxetine | 2 (0.2%) |
| Maprotiline hydrochloride | 1 (0.1%) |
| Mianserin hydrochloride | 1 (0.1%) |
| Moclobemide | 1 (0.1%) |
| Opipramol | 1 (0.1%) |
| Protriptyline | 1 (0.1%) |
| Tianeptine | 1 (0.1%) |
| Trimipramine | 1 (0.1%) |

**Table S4. Treatment-Emergent Serious Adverse Events During the Induction and Optimization/Maintenance Phases**

| System Organ Class<br>Preferred Term | Esketamine Nasal Spray<br>N=1148 |
|---|---|
| Participants with a serious adverse event | 216 (18.8%) |
| | |
| Psychiatric disorders | 55 (4.8%) |
| Depression[a] | 18 (1.6%) |
| Suicide attempt | 15 (1.3%) |
| Suicidal ideation | 11 (1.0%) |
| Anxiety | 5 (0.4%) |
| Major depression[a] | 3 (0.3%) |
| Adjustment disorder | 2 (0.2%) |
| Confusional state | 2 (0.2%) |
| Affect lability | 1 (0.1%) |
| Completed suicide | 1 (0.1%) |
| Conversion disorder | 1 (0.1%) |
| Depression suicidal | 1 (0.1%) |
| Mania | 1 (0.1%) |
| Persistent depressive disorder | 1 (0.1%) |
| Psychotic disorder | 1 (0.1%) |
| | |
| Infections and infestations | 39 (3.4%) |
| COVID-19 | 9 (0.8%) |
| Pneumonia | 7 (0.6%) |
| Cellulitis | 3 (0.3%) |
| Urinary tract infection | 3 (0.3%) |
| COVID-19 pneumonia | 2 (0.2%) |
| Cystitis | 2 (0.2%) |
| Infection | 2 (0.2%) |
| Pyelonephritis | 2 (0.2%) |
| Abscess limb | 1 (0.1%) |
| Cellulitis staphylococcal | 1 (0.1%) |
| Erysipelas | 1 (0.1%) |
| Gastroenteritis | 1 (0.1%) |
| Gastroenteritis salmonella | 1 (0.1%) |
| Gastrointestinal infection | 1 (0.1%) |
| Large intestine infection | 1 (0.1%) |
| Necrotising fasciitis | 1 (0.1%) |
| Otitis media chronic | 1 (0.1%) |
| Pharyngitis streptococcal | 1 (0.1%) |
| Postoperative wound infection | 1 (0.1%) |
| Sepsis | 1 (0.1%) |

| System Organ Class<br>Preferred Term | Esketamine Nasal Spray<br>N=1148 |
|---|---|
| Staphylococcal infection | 1 (0.1%) |
| | |
| Injury, poisoning, and procedural complications | 33 (2.9%) |
| Intentional overdose | 3 (0.3%) |
| Lower limb fracture | 3 (0.3%) |
| Ankle fracture | 2 (0.2%) |
| Fall | 2 (0.2%) |
| Meniscus injury | 2 (0.2%) |
| Overdose | 2 (0.2%) |
| Wrist fracture | 2 (0.2%) |
| Alcohol poisoning | 1 (0.1%) |
| Animal bite | 1 (0.1%) |
| Concussion | 1 (0.1%) |
| Contusion | 1 (0.1%) |
| Exposure during pregnancy | 1 (0.1%) |
| Face injury | 1 (0.1%) |
| Femur fracture | 1 (0.1%) |
| Hand fracture | 1 (0.1%) |
| Incisional hernia | 1 (0.1%) |
| Ligament rupture | 1 (0.1%) |
| Limb injury | 1 (0.1%) |
| Multiple injuries | 1 (0.1%) |
| Muscle rupture | 1 (0.1%) |
| Pelvic fracture | 1 (0.1%) |
| Radius fracture | 1 (0.1%) |
| Road traffic accident | 1 (0.1%) |
| Skin laceration | 1 (0.1%) |
| Thoracic vertebral fracture | 1 (0.1%) |
| Tibia fracture | 1 (0.1%) |
| Traumatic intracranial haemorrhage | 1 (0.1%) |
| | |
| Neoplasms benign, malignant and unspecified (incl cysts and polyps) | 18 (1.6%) |
| Breast cancer | 2 (0.2%) |
| Prostate cancer | 2 (0.2%) |
| Anal cancer | 1 (0.1%) |
| Breast neoplasm | 1 (0.1%) |
| Cholangiocarcinoma | 1 (0.1%) |
| Clear cell renal cell carcinoma | 1 (0.1%) |
| Invasive breast carcinoma | 1 (0.1%) |
| Lung adenocarcinoma | 1 (0.1%) |
| Lymphoma | 1 (0.1%) |
| Myelodysplastic syndrome | 1 (0.1%) |

| System Organ Class<br>Preferred Term | Esketamine Nasal Spray<br>N=1148 |
|---|---|
| Oesophageal squamous cell carcinoma | 1 (0.1%) |
| Ovarian germ cell teratoma | 1 (0.1%) |
| Pancreatic neuroendocrine tumour | 1 (0.1%) |
| Pituitary tumour benign | 1 (0.1%) |
| Small intestine adenocarcinoma | 1 (0.1%) |
| Squamous cell carcinoma | 1 (0.1%) |
| Transitional cell carcinoma | 1 (0.1%) |
| | |
| Gastrointestinal disorders | 17 (1.5%) |
| Haemorrhoids | 2 (0.2%) |
| Ileus | 2 (0.2%) |
| Pancreatitis acute | 2 (0.2%) |
| Umbilical hernia | 2 (0.2%) |
| Colitis | 1 (0.1%) |
| Constipation | 1 (0.1%) |
| Diarrhoea | 1 (0.1%) |
| Duodenal perforation | 1 (0.1%) |
| Gastric polyps | 1 (0.1%) |
| Hiatus hernia | 1 (0.1%) |
| Inguinal hernia | 1 (0.1%) |
| Large intestine polyp | 1 (0.1%) |
| Lower gastrointestinal haemorrhage | 1 (0.1%) |
| Pancreatitis relapsing | 1 (0.1%) |
| | |
| Respiratory, thoracic and mediastinal disorders | 17 (1.5%) |
| Asthma | 2 (0.2%) |
| Nasal polyps | 2 (0.2%) |
| Pulmonary embolism | 2 (0.2%) |
| Sleep apnoea syndrome | 2 (0.2%) |
| Bronchitis chronic | 1 (0.1%) |
| Dyspnoea | 1 (0.1%) |
| Dyspnoea exertional | 1 (0.1%) |
| Lung disorder | 1 (0.1%) |
| Nasal septum deviation | 1 (0.1%) |
| Pneumothorax spontaneous | 1 (0.1%) |
| Pulmonary oedema | 1 (0.1%) |
| Tonsillar hypertrophy | 1 (0.1%) |
| Vocal cord thickening | 1 (0.1%) |
| | |
| Cardiac disorders | 16 (1.4%) |
| Atrial fibrillation | 5 (0.4%) |
| Myocardial infarction | 4 (0.3%) |
| Acute myocardial infarction | 2 (0.2%) |

| System Organ Class<br>Preferred Term | Esketamine Nasal Spray<br>N=1148 |
|---|---|
| Coronary artery disease | 2 (0.2%) |
| Arrhythmia | 1 (0.1%) |
| Bradycardia | 1 (0.1%) |
| Coronary artery stenosis | 1 (0.1%) |
| Pericardial effusion | 1 (0.1%) |
| | |
| Renal and urinary disorders | 16 (1.4%) |
| Nephrolithiasis | 6 (0.5%) |
| Acute kidney injury | 2 (0.2%) |
| Stress urinary incontinence | 2 (0.2%) |
| Urethral stenosis | 2 (0.2%) |
| Bladder outlet obstruction | 1 (0.1%) |
| Chronic kidney disease | 1 (0.1%) |
| Renal artery stenosis | 1 (0.1%) |
| Renal failure | 1 (0.1%) |
| Urge incontinence | 1 (0.1%) |
| Urinary bladder polyp | 1 (0.1%) |
| Urinary incontinence | 1 (0.1%) |
| Urinary retention | 1 (0.1%) |
| | |
| Nervous system disorders | 15 (1.3%) |
| Headache | 3 (0.3%) |
| Carotid artery aneurysm | 2 (0.2%) |
| Cerebrovascular accident | 2 (0.2%) |
| Intracranial aneurysm | 2 (0.2%) |
| Akathisia | 1 (0.1%) |
| Dysarthria | 1 (0.1%) |
| Encephalopathy | 1 (0.1%) |
| Facial paralysis | 1 (0.1%) |
| Hemiparesis | 1 (0.1%) |
| Hemiplegia | 1 (0.1%) |
| Ischaemic stroke | 1 (0.1%) |
| Loss of consciousness | 1 (0.1%) |
| Metabolic encephalopathy | 1 (0.1%) |
| Occipital neuralgia | 1 (0.1%) |
| Seizure | 1 (0.1%) |
| Transient ischaemic attack | 1 (0.1%) |
| | |
| Hepatobiliary disorders | 14 (1.2%) |
| Cholelithiasis | 10 (0.9%) |
| Cholecystitis | 3 (0.3%) |
| Bile duct stone | 1 (0.1%) |
| Biliary obstruction | 1 (0.1%) |

| **System Organ Class**<br>**Preferred Term** | **Esketamine Nasal Spray**<br>**N=1148** |
|---|---|
| Musculoskeletal and connective tissue disorders | 14 (1.2%) |
| Back pain | 4 (0.3%) |
| Intervertebral disc protrusion | 3 (0.3%) |
| Osteoarthritis | 3 (0.3%) |
| Intervertebral disc degeneration | 2 (0.2%) |
| Rotator cuff syndrome | 2 (0.2%) |
| Arthritis | 1 (0.1%) |
| Cervical spinal stenosis | 1 (0.1%) |
| Facet joint syndrome | 1 (0.1%) |
| Intervertebral disc disorder | 1 (0.1%) |
| Spondylolisthesis | 1 (0.1%) |
| | |
| Reproductive system and breast disorders | 7 (0.6%) |
| Breast hyperplasia | 1 (0.1%) |
| Cervical dysplasia | 1 (0.1%) |
| Endometriosis | 1 (0.1%) |
| Epididymal cyst | 1 (0.1%) |
| Heavy menstrual bleeding | 1 (0.1%) |
| Rectocele | 1 (0.1%) |
| Vaginal prolapse | 1 (0.1%) |
| | |
| Metabolism and nutrition disorders | 6 (0.5%) |
| Hyperglycaemia | 2 (0.2%) |
| Dehydration | 1 (0.1%) |
| Diabetic ketoacidosis | 1 (0.1%) |
| Hypercalcaemia | 1 (0.1%) |
| Obesity | 1 (0.1%) |
| Type 2 diabetes mellitus | 1 (0.1%) |
| | |
| Vascular disorders | 5 (0.4%) |
| Aortic dissection | 1 (0.1%) |
| Circulatory collapse | 1 (0.1%) |
| Hypertensive emergency | 1 (0.1%) |
| Orthostatic hypotension | 1 (0.1%) |
| Peripheral arterial occlusive disease | 1 (0.1%) |
| | |
| General disorders and administration site conditions | 4 (0.3%) |
| Asthenia | 1 (0.1%) |
| Death | 1 (0.1%) |
| Fatigue | 1 (0.1%) |
| Pyrexia | 1 (0.1%) |

| System Organ Class<br>Preferred Term | Esketamine Nasal Spray<br>N=1148 |
|---|---|
| Ear and labyrinth disorders | 3 (0.3%) |
| Vertigo | 2 (0.2%) |
| Vertigo positional | 1 (0.1%) |
| Surgical and medical procedures | 3 (0.3%) |
| Abdominoplasty | 1 (0.1%) |
| Female sterilisation | 1 (0.1%) |
| Mammoplasty | 1 (0.1%) |
| Spinal fusion surgery | 1 (0.1%) |
| Investigations | 2 (0.2%) |
| Blood pressure diastolic increased | 1 (0.1%) |
| Myocardial necrosis marker increased | 1 (0.1%) |
| Blood and lymphatic system disorders | 1 (0.1%) |
| Thrombocytopenia | 1 (0.1%) |
| Congenital, familial, and genetic disorders | 1 (0.1%) |
| Myocardial bridging | 1 (0.1%) |
| Immune system disorders | 1 (0.1%) |
| Anaphylactic reaction | 1 (0.1%) |
| Product issues | 1 (0.1%) |
| Device breakage | 1 (0.1%) |

a. All adverse events are reported as preferred terms, coded using the Medical Dictionary for Regulatory Activities (MedDRA) version 25.1. Thus, verbatim reporting of "worsening of depression", "relapse of depression", or "hospitalization due to depression" as adverse events were coded and are reported as depression, and "worsening of major depression" or "relapse of major depression", etc. as major depression.

Note: Treatment-emergent adverse events are defined as events with onset during treatment or that were a consequence of a pre-existing condition that had worsened since baseline. Incidence is based on the number of participants experiencing at least one adverse event, not the number of events.

**Results S1. Deaths**

There were 9 (0.8%) deaths, 1 and 8 during the induction and optimization/maintenance phases, respectively), none considered by the investigator as related to esketamine. The deaths included:

- multiple injuries secondary to a bike accident: 59-year-old male, death on day 364, 6 days after last dose of esketamine
- myocardial infarction: 73-year-old female, death on day 321, 6 days after last dose of esketamine
- pneumonia: 66-year-old female, death on day 1968, 65 days after last dose of esketamine
- pneumonia: 62-year-old male, death on day 1279, 13 days after last dose of esketamine
- COVID-19/pneumonia: 66-year-old male, death on day 934, 13 days after last dose of esketamine
- COVID-19: 60-year-old male, death on day 1149, 15 days after last dose of esketamine
- COVID-19: 60-year-old male, death on day 1062, 24 days after last dose of esketamine
- reason not reported: 58-year-old male, death on day 1245, 8 days after last dose of esketamine
- completed suicide: 48-year-old male, death on day 26, 4 days after last dose of esketamine

The latter participant did not respond to esketamine (MADRS score: 35, 41, and 25 at baseline, day 15, and day 22, respectively). Considering this individual's long history of mental illness and underlying TRD, the event was reported by the site investigator as not related to esketamine.

**Table S5. Treatment-Emergent Adverse Events Leading to Discontinuation of Esketamine During the Induction and Optimization/Maintenance Phases**

| Preferred Term | Esketamine Nasal Spray N=1148 |
|---|---|
| Participants with adverse event leading to discontinuation | 72[a] (6.3%) |
| | |
| Blood pressure increased | 6 (0.5%) |
| Dissociation | 5 (0.4%) |
| Anxiety | 3 (0.3%) |
| Depression | 3 (0.3%) |
| Major depression | 3 (0.3%) |
| Mania | 3 (0.3%) |
| Fatigue | 2 (0.2%) |
| Myocardial infarction | 2 (0.2%) |
| Pneumonia | 2 (0.2%) |
| Suicidal ideation | 2 (0.2%) |
| Vertigo | 2 (0.2%) |

a. Two additional participants discontinued study drug due to "intentional overdose" and "cholangio carcinoma", respectively. However, study drug withdrawal was not selected for these two participants on the adverse events forms thus these 2 participants are not included in this table.

Note: The following treatment-emergent adverse events were reported as leading to the discontinuation of study drug for 1 (0.1%) participant each: abdominal pain, acute kidney injury, aggression, agitation, alanine aminotransferase increased, angina unstable, aortic dissection, asthma, atrial fibrillation, blood pressure systolic increased, breast neoplasm, bundle branch block left, carotid artery aneurysm, cerebrovascular accident, COVID-19, disinhibition, dizziness, dysarthria, electrocardiogram QT prolonged, electrocardiogram ST segment depression, euphoric mood, gamma-glutamyl transferase increased, haematuria, hallucination, hemiplegia, hepatic enzyme increased, hypersomnia, hypertensive emergency, hypoaesthesia, hypomania, intracranial aneurysm, irritability, ischaemic stroke, lacunar infarction, loss of consciousness, lower gastrointestinal haemorrhage, lymphoma, nasal congestion, nausea, oesophageal squamous cell carcinoma, overdose, paranoia, pelvic fracture, renal tubular necrosis, retrograde amnesia, rhabdomyolysis, seizure, suicide attempt, transient ischaemic attack, type 2 diabetes mellitus, urinary incontinence, vertebral artery occlusion, visual impairment, vomiting.

An adverse event that started in the induction or optimization/maintenance phase and resulted in discontinuation of study drug is counted as treatment-emergent in this table.

**Figure S4. Event Rate of Treatment-Emergent Adverse Events of Dissociation Persisting Beyond 2 Hours**

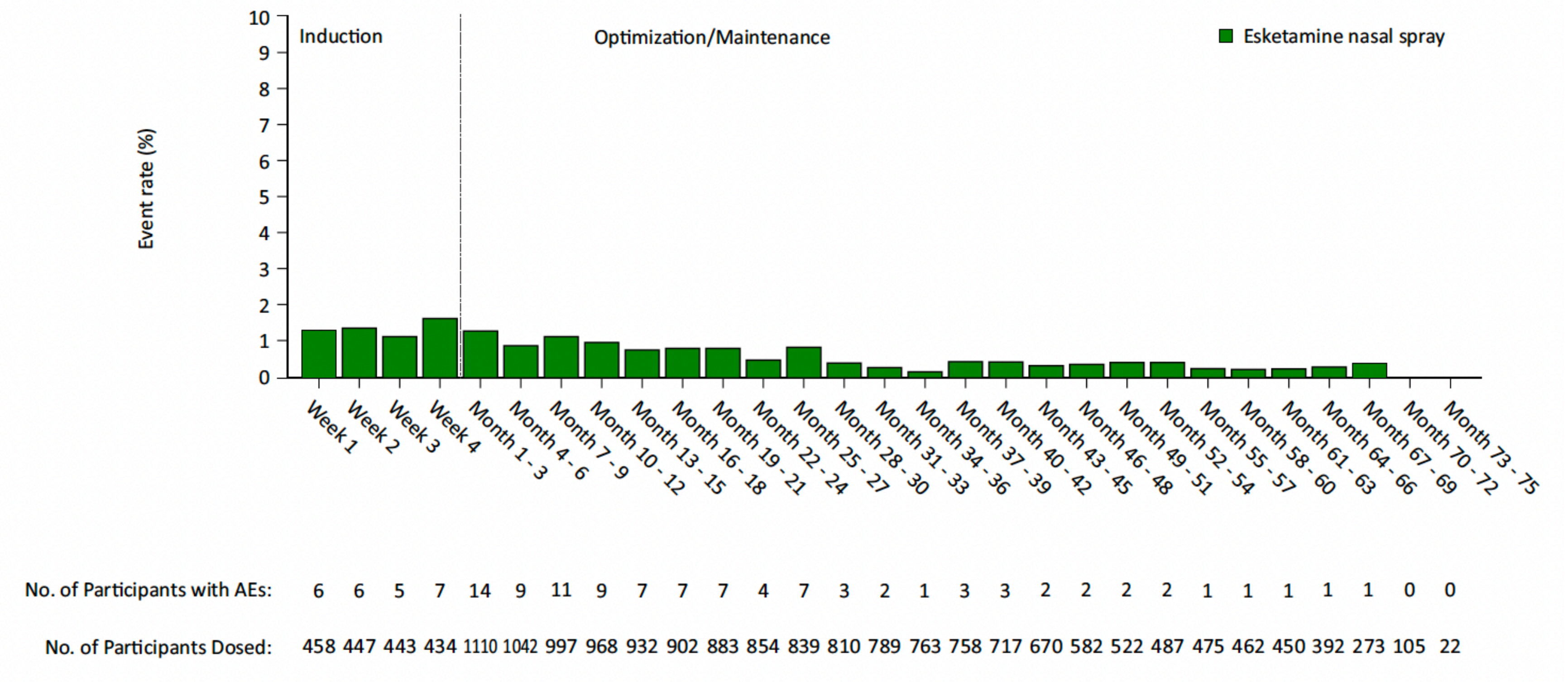


AE = adverse event.

Notes: Adverse events rate = number of participants dosed and with treatment-emergent adverse events / number of participants dosed within interval. Study week (or month) was defined using the phase study day by every 7 days (or 28 days). The intervals with the number of participants <10 are not presented. Data are plotted per week in the induction phase and quarterly in the optimization/maintenance phase.

**Figure S5. Event Rate of Treatment-Emergent Hepatic Adverse Events**

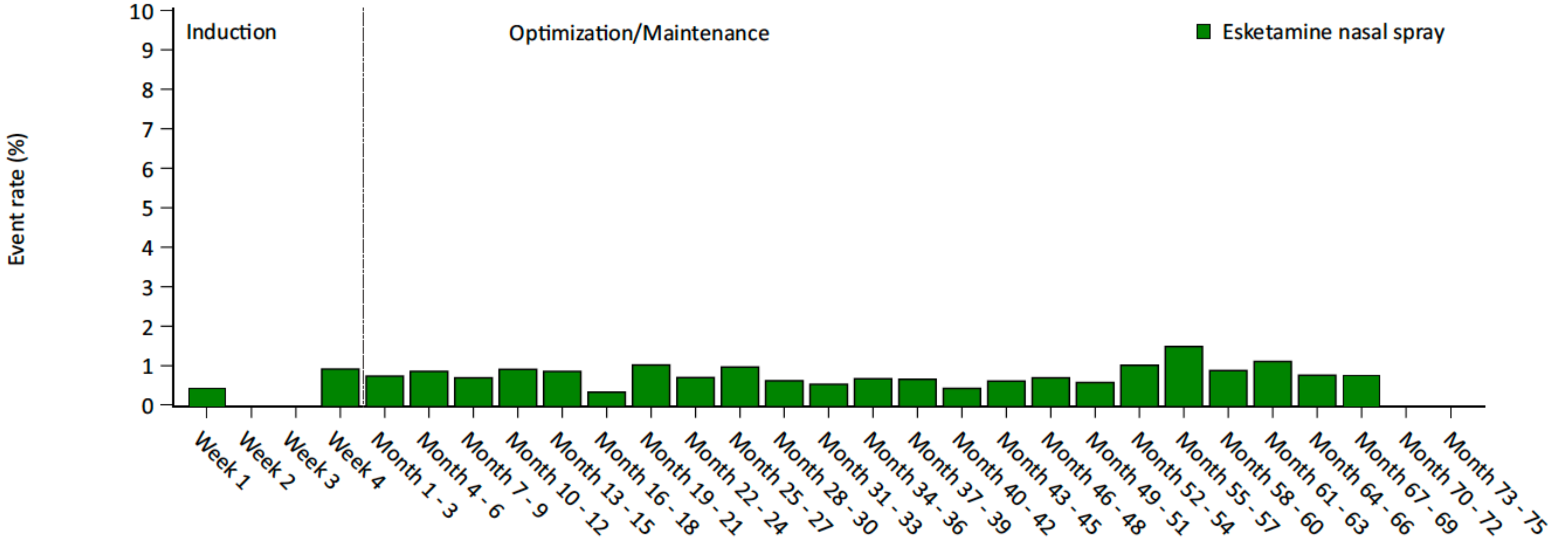


| | | | | | | | | | | | | | | | | | | | | | | | | | | | | | |
|---|---|---|---|---|---|---|---|---|---|---|---|---|---|---|---|---|---|---|---|---|---|---|---|---|---|---|---|---|---|
| No. of Participants with AEs: | 2 | 0 | 0 | 4 | 8 | 9 | 7 | 9 | 8 | 3 | 9 | 6 | 8 | 5 | 4 | 5 | 5 | 3 | 4 | 4 | 3 | 5 | 7 | 4 | 5 | 3 | 2 | 0 | 0 |
| No. of Participants Dosed: | 458 | 447 | 443 | 434 | 1110 | 1042 | 997 | 968 | 932 | 902 | 883 | 854 | 839 | 810 | 789 | 763 | 758 | 717 | 670 | 582 | 522 | 487 | 475 | 462 | 450 | 392 | 273 | 105 | 22 |

AE = adverse event.
Notes: Adverse events rate = number of participants dosed and with treatment-emergent adverse events in interval / number of participants dosed within interval.
Study week (or month) was defined using the phase study day by every 7 days (or 28 days).
The intervals with the number of participants <10 are not presented. Data are plotted per week in the induction phase and quarterly in the optimization/maintenance phase.

## Figure S6. Incidence of Increased Blood Pressure Adverse Events Over Time

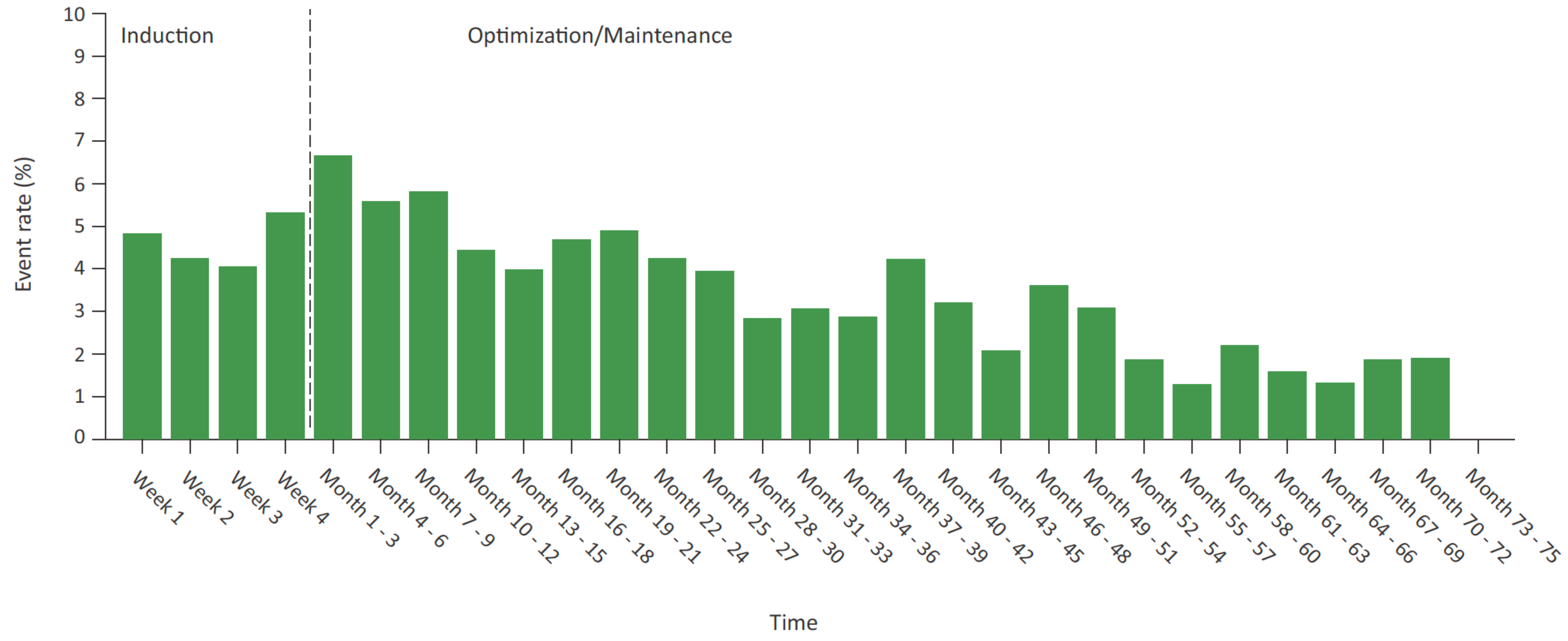


| | | | | | | | | | | | | | | | | | | | | | | | | | | | | | |
|---|---|---|---|---|---|---|---|---|---|---|---|---|---|---|---|---|---|---|---|---|---|---|---|---|---|---|---|---|---|
| No. of Participants with AEs | | | | | | | | | | | | | | | | | | | | | | | | | | | | | |
| Esketamine Nasal Spray | 22 | 19 | 18 | 23 | 74 | 58 | 58 | 43 | 37 | 42 | 43 | 36 | 33 | 23 | 24 | 22 | 32 | 23 | 14 | 21 | 16 | 9 | 6 | 10 | 7 | 5 | 5 | 2 | 0 |
| No. of Participants Dosed | | | | | | | | | | | | | | | | | | | | | | | | | | | | | |
| Esketamine Nasal Spray | 458 | 447 | 443 | 434 | 1110 | 1042 | 997 | 968 | 932 | 902 | 883 | 854 | 839 | 810 | 789 | 763 | 758 | 717 | 670 | 582 | 522 | 487 | 475 | 462 | 450 | 392 | 273 | 105 | 22 |

AE = adverse event.
Adverse events rate is the number of participants dosed and with treatment-emergent adverse event(s) in interval / number of participants dosed within interval. Study week (or month) was defined using the phase study day by every 7 days (or 28 days). The intervals with the number of participants <10 are not presented. Data are plotted per week in the induction phase and quarterly in the optimization/maintenance phase.
Data for the induction phase are reported elsewhere [Zaki et al., 2023].


Zaki N, Chen LN, Lane R, et al. Long-term safety and maintenance of response with esketamine nasal spray in participants with treatment-resistant depression: interim results of the SUSTAIN-3 study. Neuropsychopharmacology. 2023;48(8):1225-1233. doi: 10.1038/s41386-023-01577-5

## Results S2. Results of Cognitive Assessments

Performance on most cognitive tests remained stable throughout optimization/maintenance for participants <65 years and participants ≥65 years, including on tests of verbal and working memory, and executive function (Figure S9 through Figure S15).

Reaction times slowed during optimization/maintenance, based on arithmetic mean and mean change from study baseline scores (Figure S7 and Figure S8). Cohen's d statistic indicated that the slowing reached a magnitude suggestive of a small effect size on Detection (DET) (simple reaction time) for participants <65 years beginning at week 208 (Table S6). However, participants' reaction time performance fluctuated considerably across timepoints (i.e., intraindividual variability). Of the 604 participants who attained a Reliable Change Index (RCI) score < -1.96 at some point during optimization/maintenance, 21 had RCI scores that continued < -1.96 throughout subsequent timepoints whereas 550 participants subsequently attained RCI score(s) > -1.96. Thirty-three participants attained RCI < -1.96 at final assessment during optimization/maintenance (Table S7). Similarly, Cohen's d statistic indicated that slowing reached a magnitude suggestive of a small effect size on DET for participants ≥ 65 years at weeks 64-124 (Table S6). Similar to the participants <65 years, older participants exhibited intraindividual variability in DET performance. Of the 79 participants ≥65 years who attained an RCI score < -1.96 at some point during optimization/maintenance, 9 had RCI scores that continued < -1.96 across subsequent timepoints whereas 66 participants attained RCI scores > -1.96 at subsequent timepoints. Four participants attained RCI < -1.96 at their final assessment during optimization/maintenance (Table S7).

For choice reaction time (Identification [IDN]), Cohen's d statistic indicated that slowing reached a magnitude suggestive of a small effect size for participants <65 years at weeks 184-292 (Table S6). Of 573 participants who attained RCI < -1.96 on IDN during optimization/maintenance, 21 continued to perform with RCI < -1.96 across subsequent timepoints whereas 528 participants returned to RCI value(s) > -1.96 at subsequent assessments; 24 participants attained RCI < -1.96 at the final assessment during optimization/maintenance (Table S7). Similarly, Cohen's d statistic indicated that slowing on IDN reached a magnitude suggestive of a small effect size on IDN for participants ≥65 years at weeks 40-124 (Table S6).

There was considerable intraindividual variability among participants ≥65 years on IDN RCI scores across timepoints. Of 78 participants who attained RCI < -1.96 at some point during optimization/maintenance, 8 continued with RCI < -1.96 throughout subsequent timepoints whereas 66 participants returned to performance levels > -1.96. Three participants attained RCI < -1.96 at final assessment during optimization/maintenance (Table S7).

## Figure S7. Detection – Attention (Simple Reaction Time) by Age Group Over the Induction and Optimization/Maintenance Phases of SUSTAIN-3

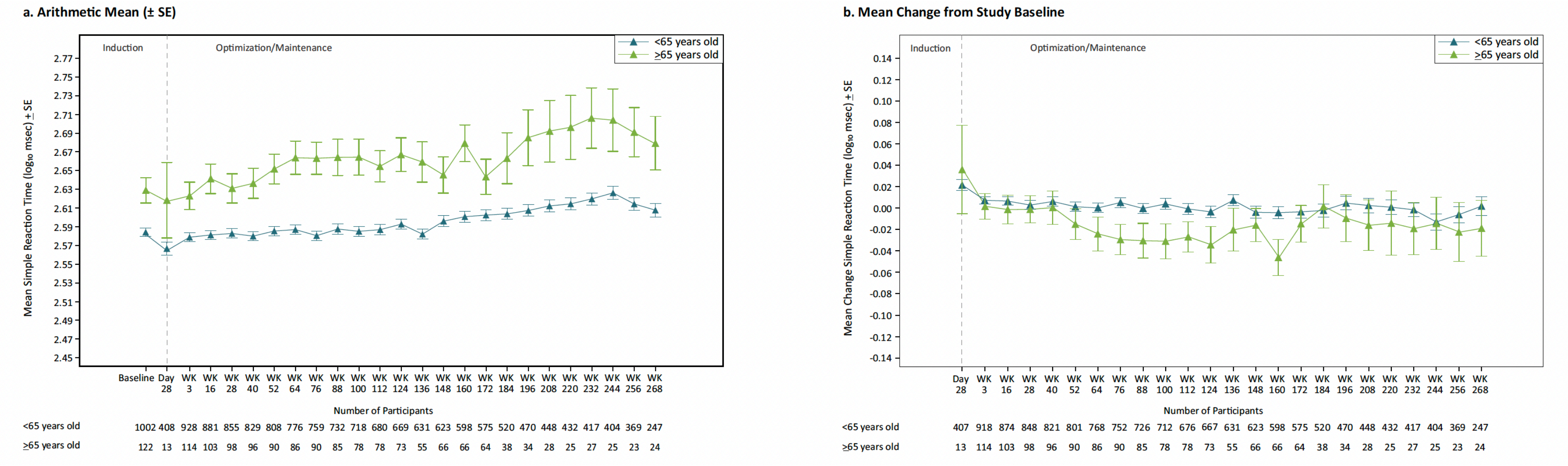


Notes: For participants who entered the study at the induction phase, study baseline was defined as the last observation prior to or on the start date of the induction phase. For participants who entered the study at the optimization/maintenance phase, study baseline was defined as the last observation prior to or on the start date of the optimization/maintenance phase. Lower arithmetic mean scores represent better performance; for mean change from baseline calculations, higher mean change from baseline represents better performance.

**Figure S8. Identification – Attention (Choice Reaction Time) by Age Group Over the Induction and Optimization/Maintenance Phases of SUSTAIN-3**

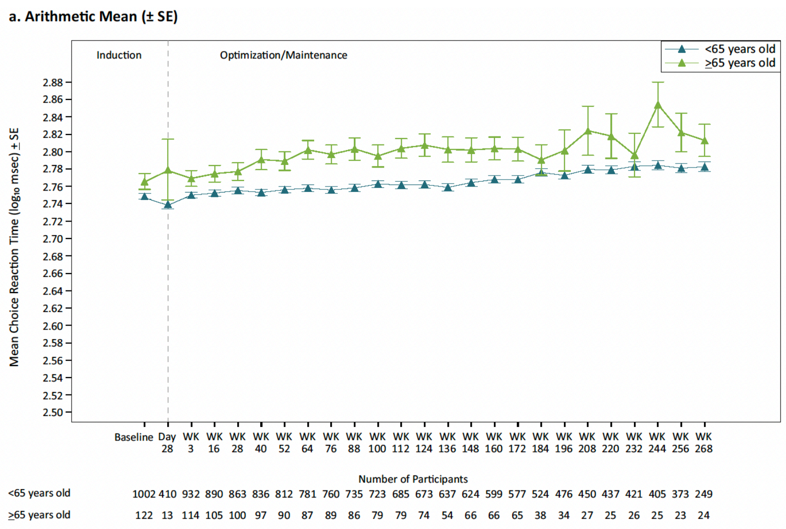


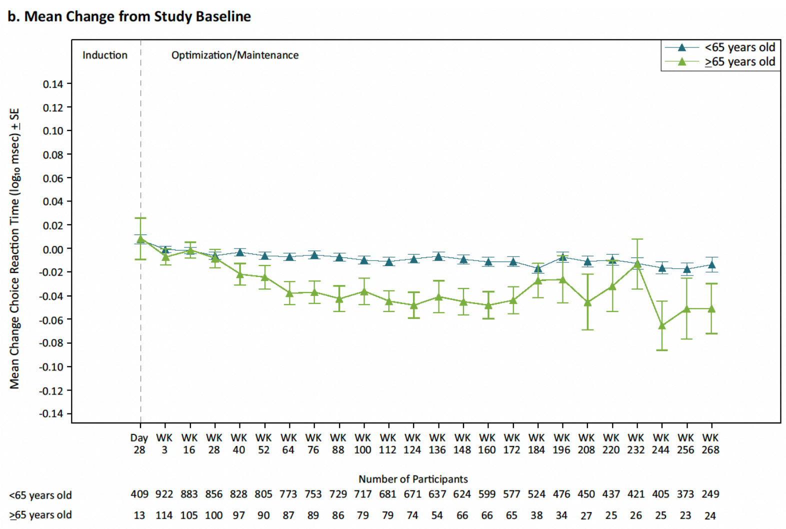


Notes: For participants who entered the study at the induction phase, study baseline was defined as the last observation prior to or on the start date of the induction phase. For participants who entered the study at the optimization/maintenance phase, study baseline was defined as the last observation prior to or on the start date of the optimization/maintenance phase. Lower arithmetic mean scores represent better performance; for mean change from baseline calculations, higher mean change from baseline represents better performance.

## Figure S9. One Card Learning – Visual Learning by Age Group Over the Induction and Optimization/Maintenance Phases of SUSTAIN-3

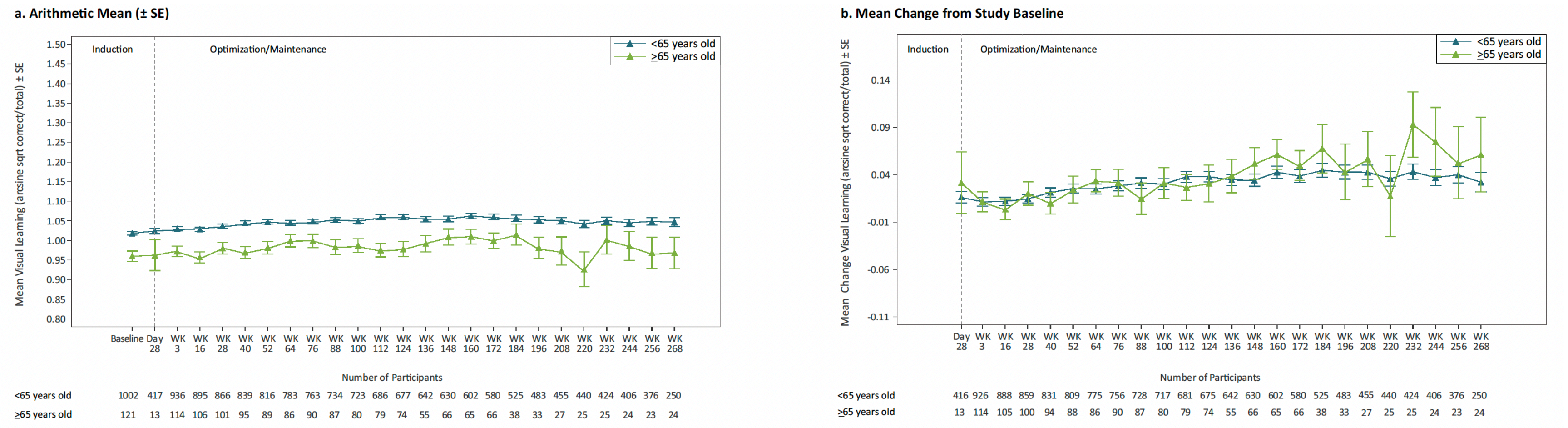


Notes: For participants who entered the study at the induction phase, study baseline was defined as the last observation prior to or on the start date of the induction phase. For participants who entered the study at the optimization/maintenance phase, study baseline was defined as the last observation prior to or on the start date of the optimization/maintenance phase. Higher arithmetic mean scores and higher mean change from baseline represent better performance.

## Figure S10. One Back Test – Working Memory by Age Group Over the Induction and Optimization/Maintenance Phases of SUSTAIN-3

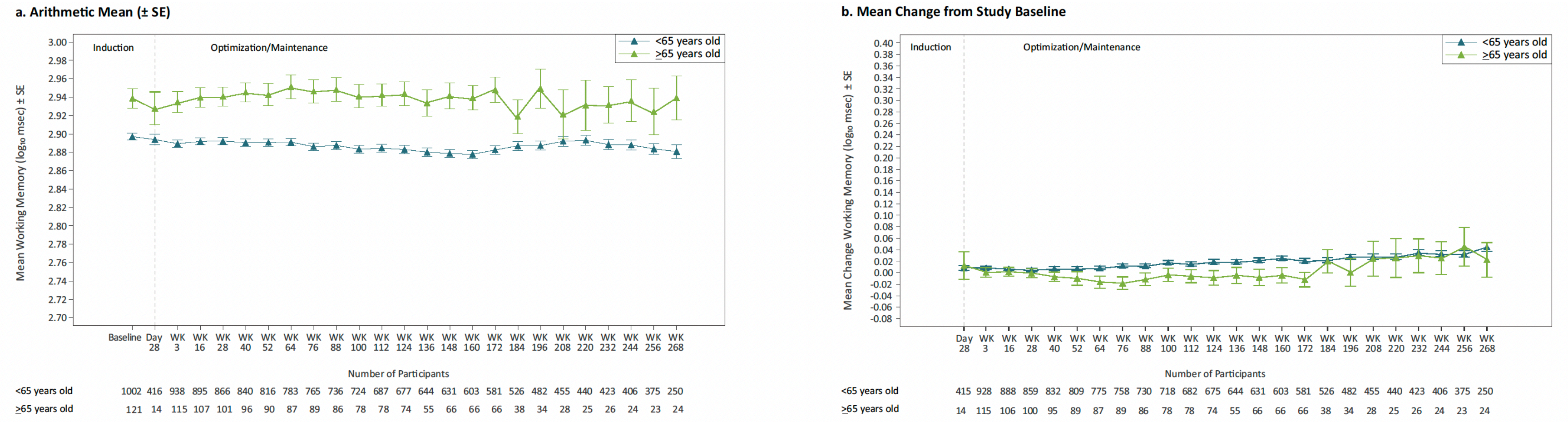


Notes: For participants who entered the study at the induction phase, study baseline was defined as the last observation prior to or on the start date of the induction phase. For participants who entered the study at the optimization/maintenance phase, study baseline was defined as the last observation prior to or on the start date of the optimization/maintenance phase. Lower arithmetic mean scores represent better performance; for mean change from baseline calculations, higher mean change from baseline represents better performance.

**Figure S11.** **Groton Maze Learning Test – Executive Function by Age Group Over the Induction and Optimization/Maintenance Phases of SUSTAIN-3**

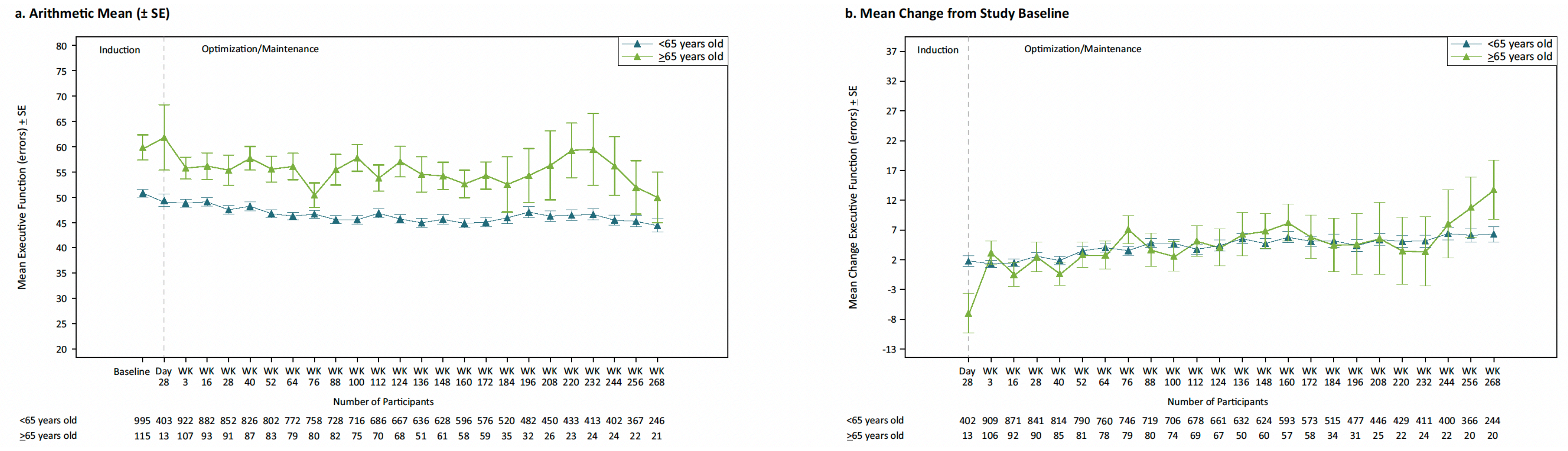


Notes: For participants who entered the study at the induction phase, study baseline was defined as the last observation prior to or on the start date of the induction phase. For participants who entered the study at the optimization/maintenance phase, study baseline was defined as the last observation prior to or on the start date of the optimization/maintenance phase. Lower arithmetic mean scores represent better performance; for mean change from baseline calculations, higher mean change from baseline represents better performance.

## Figure S12. HVLT-R Total Recall by Age Group Over the Induction and Optimization/Maintenance Phases of SUSTAIN-3

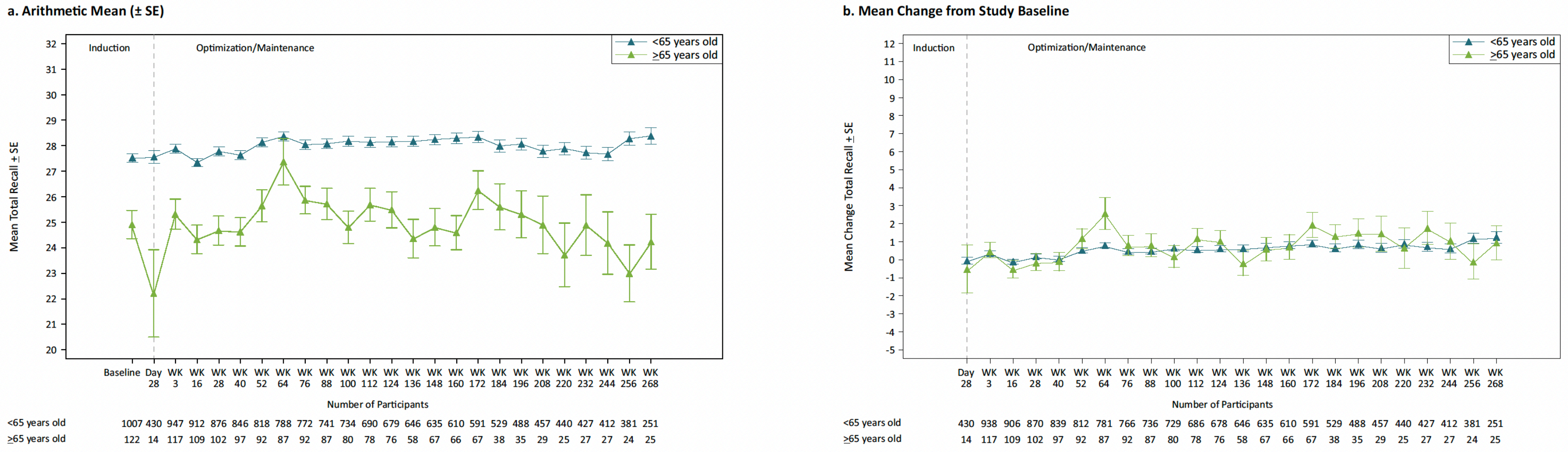


Notes: For participants who entered the study at the induction phase, study baseline was defined as the last observation prior to or on the start date of the induction phase. For participants who entered the study at the optimization/maintenance phase, study baseline was defined as the last observation prior to or on the start date of the optimization/maintenance phase. Higher arithmetic mean score and higher mean change from baseline represent better performance.

## Figure S13. HVLT-R Delayed Recall by Age Group Over the Induction and Optimization/Maintenance Phases of SUSTAIN-3

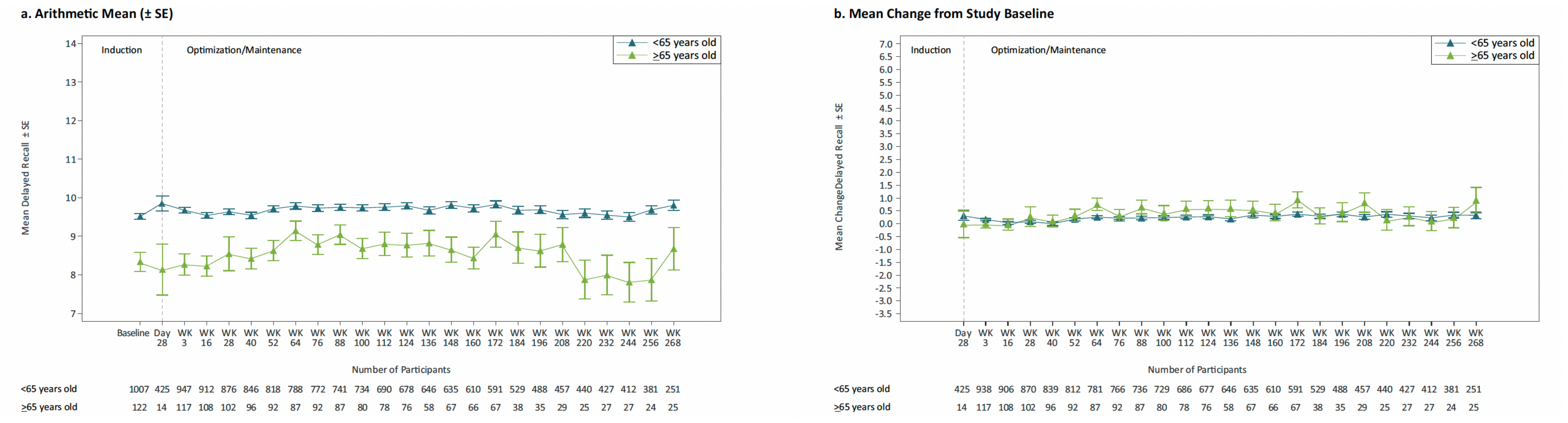


Notes: For participants who entered the study at the induction phase, study baseline was defined as the last observation prior to or on the start date of the induction phase. For participants who entered the study at the optimization/maintenance phase, study baseline was defined as the last observation prior to or on the start date of the optimization/maintenance phase. Higher arithmetic mean score and higher mean change from baseline represent better performance.

## Figure S14. HVLT-R Total Number of True Positives by Age Group Over the Induction and Optimization/Maintenance Phases of SUSTAIN-3

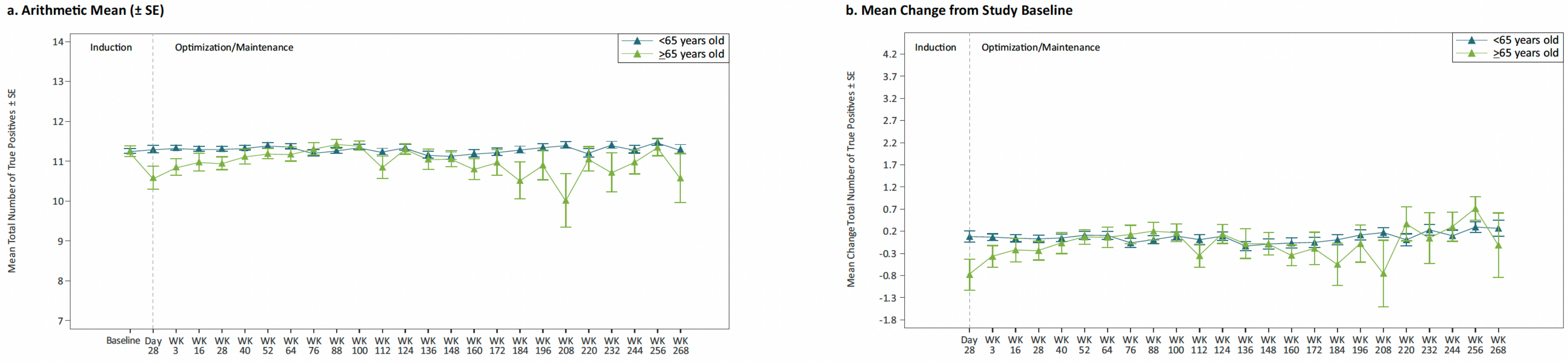


Number of Participants

| | Baseline | Day 28 | WK 3 | WK 16 | WK 28 | WK 40 | WK 52 | WK 64 | WK 76 | WK 88 | WK 100 | WK 112 | WK 124 | WK 136 | WK 148 | WK 160 | WK 172 | WK 184 | WK 196 | WK 208 | WK 220 | WK 232 | WK 244 | WK 256 | WK 268 |
|---|---|---|---|---|---|---|---|---|---|---|---|---|---|---|---|---|---|---|---|---|---|---|---|---|---|
| <65 years old | 1007 | 429 | 946 | 912 | 876 | 846 | 817 | 788 | 772 | 741 | 734 | 690 | 678 | 646 | 635 | 610 | 591 | 529 | 488 | 457 | 440 | 427 | 412 | 381 | 251 |
| ≥65 years old | 122 | 14 | 117 | 109 | 102 | 97 | 92 | 87 | 92 | 87 | 80 | 78 | 76 | 58 | 67 | 66 | 67 | 38 | 35 | 29 | 25 | 27 | 27 | 24 | 25 |

Number of Participants

| | Day 28 | WK 3 | WK 16 | WK 28 | WK 40 | WK 52 | WK 64 | WK 76 | WK 88 | WK 100 | WK 112 | WK 124 | WK 136 | WK 148 | WK 160 | WK 172 | WK 184 | WK 196 | WK 208 | WK 220 | WK 232 | WK 244 | WK 256 | WK 268 |
|---|---|---|---|---|---|---|---|---|---|---|---|---|---|---|---|---|---|---|---|---|---|---|---|---|
| <65 years old | 429 | 937 | 906 | 870 | 839 | 811 | 781 | 766 | 736 | 729 | 686 | 677 | 646 | 635 | 610 | 591 | 529 | 488 | 457 | 440 | 427 | 412 | 381 | 251 |
| ≥65 years old | 14 | 117 | 109 | 102 | 97 | 92 | 87 | 92 | 87 | 80 | 78 | 76 | 58 | 67 | 66 | 67 | 38 | 35 | 29 | 25 | 27 | 27 | 24 | 25 |

Notes: For participants who entered the study at the induction phase, study baseline was defined as the last observation prior to or on the start date of the induction phase. For participants who entered the study at the optimization/maintenance phase, study baseline was defined as the last observation prior to or on the start date of the optimization/maintenance phase. Higher arithmetic mean score and higher mean change from baseline represent better performance.

**Figure S15. HVLT-R Recognition Discrimination Index by Age Group Over the Induction and Optimization/Maintenance Phases of SUSTAIN-3**

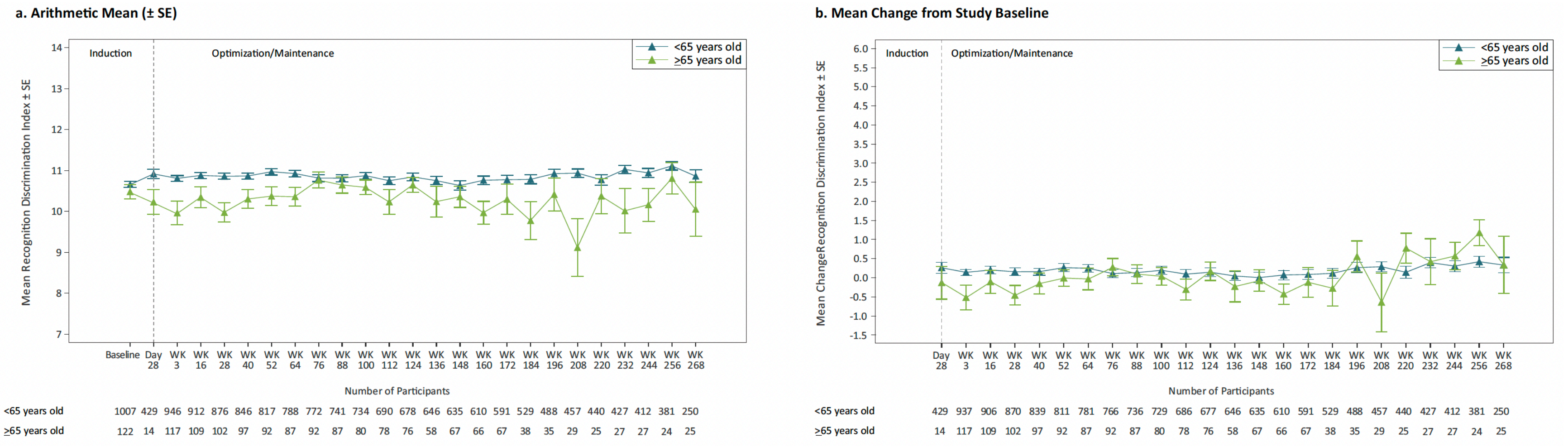


Notes: For participants who entered the study at the induction phase, study baseline was defined as the last observation prior to or on the start date of the induction phase. For participants who entered the study at the optimization/maintenance phase, study baseline was defined as the last observation prior to or on the start date of the optimization/maintenance phase. Higher arithmetic mean score and higher mean change from baseline represent better performance.

**Table S6. Effect Size (Cohen's d) for Cogstate Tests Scored on Basis of Processing Speed by Age Subgroup**

| Cogstate Test<br>Age Subgroup | Visit | N | Mean | SD | Cohen's d |
|---|---|---|---|---|---|
| Detection Speed of Performance | | | | | |
| <65 years | Day 28 (IND) | 408 | 2.57 | 0.141 | -0.12 |
| | Endpoint (IND) | 408 | 2.57 | 0.141 | -0.12 |
| | Week 3 (OP/MA) | 928 | 2.58 | 0.141 | -0.04 |
| | Week 16 (OP/MA) | 881 | 2.58 | 0.141 | -0.02 |
| | Week 28 (OP/MA) | 855 | 2.58 | 0.146 | -0.01 |
| | Week 40 (OP/MA) | 829 | 2.58 | 0.137 | -0.03 |
| | Week 52 (OP/MA) | 808 | 2.59 | 0.142 | 0.01 |
| | Week 64 (OP/MA) | 776 | 2.59 | 0.140 | 0.02 |
| | Week 76 (OP/MA) | 759 | 2.58 | 0.137 | -0.03 |
| | Week 88 (OP/MA) | 732 | 2.59 | 0.144 | 0.02 |
| | Week 100 (OP/MA) | 718 | 2.59 | 0.145 | 0.01 |
| | Week 112 (OP/MA) | 680 | 2.59 | 0.140 | 0.02 |
| | Week 124 (OP/MA) | 669 | 2.59 | 0.142 | 0.06 |
| | Week 136 (OP/MA) | 631 | 2.58 | 0.133 | -0.02 |
| | Week 148 (OP/MA) | 623 | 2.60 | 0.144 | 0.08 |
| | Week 160 (OP/MA) | 598 | 2.60 | 0.141 | 0.12 |
| | Week 172 (OP/MA) | 575 | 2.60 | 0.140 | 0.13 |
| | Week 184 (OP/MA) | 520 | 2.60 | 0.133 | 0.15 |
| | Week 196 (OP/MA) | 470 | 2.61 | 0.133 | 0.18 |
| | Week 208 (OP/MA) | 448 | 2.61 | 0.136 | 0.21 |
| | Week 220 (OP/MA) | 432 | 2.61 | 0.132 | 0.23 |
| | Week 232 (OP/MA) | 417 | 2.62 | 0.130 | 0.27 |
| | Week 244 (OP/MA) | 404 | 2.63 | 0.145 | 0.29 |
| | Week 256 (OP/MA) | 369 | 2.61 | 0.131 | 0.23 |
| | Week 268 (OP/MA) | 247 | 2.61 | 0.114 | 0.21 |
| | Week 280 (OP/MA) | 126 | 2.61 | 0.120 | 0.20 |
| | Week 292 (OP/MA) | 33 | 2.58 | 0.136 | 0.00 |
| | Week 304 (OP/MA) | 8 | 2.58 | 0.160 | -0.04 |
| | Week 316 (OP/MA) | 1 | 2.50 | | |
| | Endpoint (OP/MA) | 975 | 2.59 | 0.137 | 0.05 |
| ≥65 years | Day 28 (IND) | 13 | 2.62 | 0.145 | -0.07 |
| | Endpoint (IND) | 13 | 2.62 | 0.145 | -0.07 |
| | Week 3 (OP/MA) | 114 | 2.62 | 0.156 | -0.04 |
| | Week 16 (OP/MA) | 103 | 2.64 | 0.160 | 0.08 |
| | Week 28 (OP/MA) | 98 | 2.63 | 0.154 | 0.01 |
| | Week 40 (OP/MA) | 96 | 2.64 | 0.157 | 0.05 |
| | Week 52 (OP/MA) | 90 | 2.65 | 0.150 | 0.15 |

| Cogstate Test<br>Age Subgroup | Visit | N | Mean | SD | Cohen's d |
|---|---|---|---|---|---|
| | Week 64 (OP/MA) | 86 | 2.66 | 0.165 | 0.21 |
| | Week 76 (OP/MA) | 90 | 2.66 | 0.163 | 0.21 |
| | Week 88 (OP/MA) | 85 | 2.66 | 0.178 | 0.20 |
| | Week 100 (OP/MA) | 78 | 2.66 | 0.171 | 0.21 |
| | Week 112 (OP/MA) | 78 | 2.65 | 0.147 | 0.17 |
| | Week 124 (OP/MA) | 73 | 2.67 | 0.154 | 0.25 |
| | Week 136 (OP/MA) | 55 | 2.66 | 0.160 | 0.19 |
| | Week 148 (OP/MA) | 66 | 2.65 | 0.158 | 0.10 |
| | Week 160 (OP/MA) | 66 | 2.68 | 0.159 | 0.32 |
| | Week 172 (OP/MA) | 64 | 2.64 | 0.150 | 0.10 |
| | Week 184 (OP/MA) | 38 | 2.66 | 0.167 | 0.20 |
| | Week 196 (OP/MA) | 34 | 2.69 | 0.174 | 0.32 |
| | Week 208 (OP/MA) | 28 | 2.69 | 0.174 | 0.36 |
| | Week 220 (OP/MA) | 25 | 2.70 | 0.171 | 0.39 |
| | Week 232 (OP/MA) | 27 | 2.71 | 0.167 | 0.46 |
| | Week 244 (OP/MA) | 25 | 2.70 | 0.167 | 0.45 |
| | Week 256 (OP/MA) | 23 | 2.69 | 0.126 | 0.49 |
| | Week 268 (OP/MA) | 24 | 2.68 | 0.140 | 0.36 |
| | Week 280 (OP/MA) | 17 | 2.67 | 0.148 | 0.25 |
| | Week 292 (OP/MA) | 2 | 2.66 | 0.112 | 0.30 |
| | Endpoint (OP/MA) | 120 | 2.64 | 0.151 | 0.04 |
| Identification Speed of Performance | | | | | |
| <65 years | Day 28 (IND) | 410 | 2.74 | 0.106 | -0.08 |
| | Endpoint (IND) | 410 | 2.74 | 0.107 | -0.08 |
| | Week 3 (OP/MA) | 932 | 2.75 | 0.104 | 0.01 |
| | Week 16 (OP/MA) | 890 | 2.75 | 0.103 | 0.04 |
| | Week 28 (OP/MA) | 863 | 2.76 | 0.111 | 0.06 |
| | Week 40 (OP/MA) | 836 | 2.75 | 0.104 | 0.04 |
| | Week 52 (OP/MA) | 812 | 2.76 | 0.110 | 0.07 |
| | Week 64 (OP/MA) | 781 | 2.76 | 0.105 | 0.09 |
| | Week 76 (OP/MA) | 760 | 2.76 | 0.109 | 0.07 |
| | Week 88 (OP/MA) | 735 | 2.76 | 0.110 | 0.09 |
| | Week 100 (OP/MA) | 723 | 2.76 | 0.109 | 0.13 |
| | Week 112 (OP/MA) | 685 | 2.76 | 0.109 | 0.12 |
| | Week 124 (OP/MA) | 673 | 2.76 | 0.110 | 0.13 |
| | Week 136 (OP/MA) | 637 | 2.76 | 0.104 | 0.10 |
| | Week 148 (OP/MA) | 624 | 2.76 | 0.106 | 0.15 |
| | Week 160 (OP/MA) | 599 | 2.77 | 0.105 | 0.18 |
| | Week 172 (OP/MA) | 577 | 2.77 | 0.104 | 0.19 |
| | Week 184 (OP/MA) | 524 | 2.78 | 0.099 | 0.28 |
| | Week 196 (OP/MA) | 476 | 2.77 | 0.098 | 0.25 |

| Cogstate Test<br>Age Subgroup | Visit | N | Mean | SD | Cohen's d |
|---|---|---|---|---|---|
| | Week 208 (OP/MA) | 450 | 2.78 | 0.102 | 0.30 |
| | Week 220 (OP/MA) | 437 | 2.78 | 0.098 | 0.31 |
| | Week 232 (OP/MA) | 421 | 2.78 | 0.100 | 0.35 |
| | Week 244 (OP/MA) | 405 | 2.78 | 0.104 | 0.34 |
| | Week 256 (OP/MA) | 373 | 2.78 | 0.100 | 0.33 |
| | Week 268 (OP/MA) | 249 | 2.78 | 0.091 | 0.37 |
| | Week 280 (OP/MA) | 128 | 2.78 | 0.097 | 0.33 |
| | Week 292 (OP/MA) | 31 | 2.80 | 0.118 | 0.41 |
| | Week 304 (OP/MA) | 8 | 2.77 | 0.114 | 0.17 |
| | Week 316 (OP/MA) | 1 | 2.63 | | |
| | Endpoint (OP/MA) | 974 | 2.77 | 0.107 | 0.17 |
| ≥65 years | Day 28 (IND) | 13 | 2.78 | 0.126 | 0.11 |
| | Endpoint (IND) | 13 | 2.78 | 0.126 | 0.11 |
| | Week 3 (OP/MA) | 114 | 2.77 | 0.096 | 0.04 |
| | Week 16 (OP/MA) | 105 | 2.77 | 0.099 | 0.09 |
| | Week 28 (OP/MA) | 100 | 2.78 | 0.102 | 0.11 |
| | Week 40 (OP/MA) | 97 | 2.79 | 0.114 | 0.22 |
| | Week 52 (OP/MA) | 90 | 2.79 | 0.102 | 0.23 |
| | Week 64 (OP/MA) | 87 | 2.80 | 0.100 | 0.36 |
| | Week 76 (OP/MA) | 89 | 2.80 | 0.102 | 0.30 |
| | Week 88 (OP/MA) | 86 | 2.80 | 0.120 | 0.31 |
| | Week 100 (OP/MA) | 79 | 2.79 | 0.113 | 0.26 |
| | Week 112 (OP/MA) | 79 | 2.80 | 0.100 | 0.38 |
| | Week 124 (OP/MA) | 74 | 2.81 | 0.113 | 0.37 |
| | Week 136 (OP/MA) | 54 | 2.80 | 0.107 | 0.35 |
| | Week 148 (OP/MA) | 66 | 2.80 | 0.112 | 0.32 |
| | Week 160 (OP/MA) | 66 | 2.80 | 0.107 | 0.36 |
| | Week 172 (OP/MA) | 65 | 2.80 | 0.109 | 0.34 |
| | Week 184 (OP/MA) | 38 | 2.79 | 0.107 | 0.23 |
| | Week 196 (OP/MA) | 34 | 2.80 | 0.138 | 0.26 |
| | Week 208 (OP/MA) | 27 | 2.82 | 0.147 | 0.40 |
| | Week 220 (OP/MA) | 25 | 2.82 | 0.129 | 0.41 |
| | Week 232 (OP/MA) | 26 | 2.80 | 0.128 | 0.24 |
| | Week 244 (OP/MA) | 25 | 2.85 | 0.129 | 0.69 |
| | Week 256 (OP/MA) | 23 | 2.82 | 0.106 | 0.53 |
| | Week 268 (OP/MA) | 24 | 2.81 | 0.090 | 0.52 |
| | Week 280 (OP/MA) | 17 | 2.80 | 0.120 | 0.29 |
| | Week 292 (OP/MA) | 2 | 2.81 | 0.048 | 0.84 |
| | Endpoint (OP/MA) | 120 | 2.79 | 0.114 | 0.21 |

IND = induction; OP/MA = optimization/maintenance.
Note: Cohen's d is calculated relative to baseline: 2.58 and 2.63 for detection and 2.75 and 2.77 for identification for the <65 and ≥65 age groups, respectively.

## Table S7. RCI Scores for Detection and Identification During Optimization/Maintenance by Age Subgroup (Number of Participants)

| Age Subgroup | Total | Reached RCI < -1.96 | Stayed RCI < -1.96 | Reached RCI < -1.96 at Last Visit | Jumped Across RCI -1.96 | Reached RCI > 1.96 | Stayed RCI > 1.96 | Reached RCI > 1.96 at Last Visit | Jumped Across RCI 1.96 |
|---|---|---|---|---|---|---|---|---|---|
| < 65 years | | | | | | | | | |
| DET | 965 | 604 | 21 | 33 | 550 | 481 | 48 | 20 | 413 |
| IDN | 964 | 573 | 21 | 24 | 528 | 391 | 31 | 10 | 350 |
| ≥ 65 years | | | | | | | | | |
| DET | 120 | 79 | 9 | 4 | 66 | 59 | 6 | 3 | 50 |
| IDN | 120 | 78 | 8 | 3 | 67 | 38 | 1 | 1 | 36 |

DET = Detection; IDN = Identification; RCI = Reliable Change Index.

Notes: Stayed RCI < -1.96, reached RCI < -1.96 at the last visit, and jumped across RCI < -1.96 are mutually exclusive subsets of reached RCI < -1.96. Stayed RCI >1.96, reached RCI >1.96 at the last visit, and jumped across RCI 1.96 are mutually exclusive subsets of reached RCI >1.96.

The Reliable Change Index (e.g., Jacobsen et al., 1984) is a psychometric calculation that provides threshold values which represent the magnitude of change on a measurement that an individual needs to exhibit to conclude that the observed change exceeds the extent of change likely attributable to measurement error. RCI ≥ 1.96 or ≤ -1.96 is considered a meaningful change from baseline (i.e., -1.96 indicates test performance was worse than at baseline and 1.96, better than at baseline).

Jacobson NS, Follette WC, Revenstorf, D. Psychotherapy outcome research: Methods for reporting variability and evaluating clinical significance. Behavior Therapy 1984;15:336–352.

**Figure S16. Incidence of Columbia-Suicide Severity Rating Scale (C-SSRS) Scores Over Time**

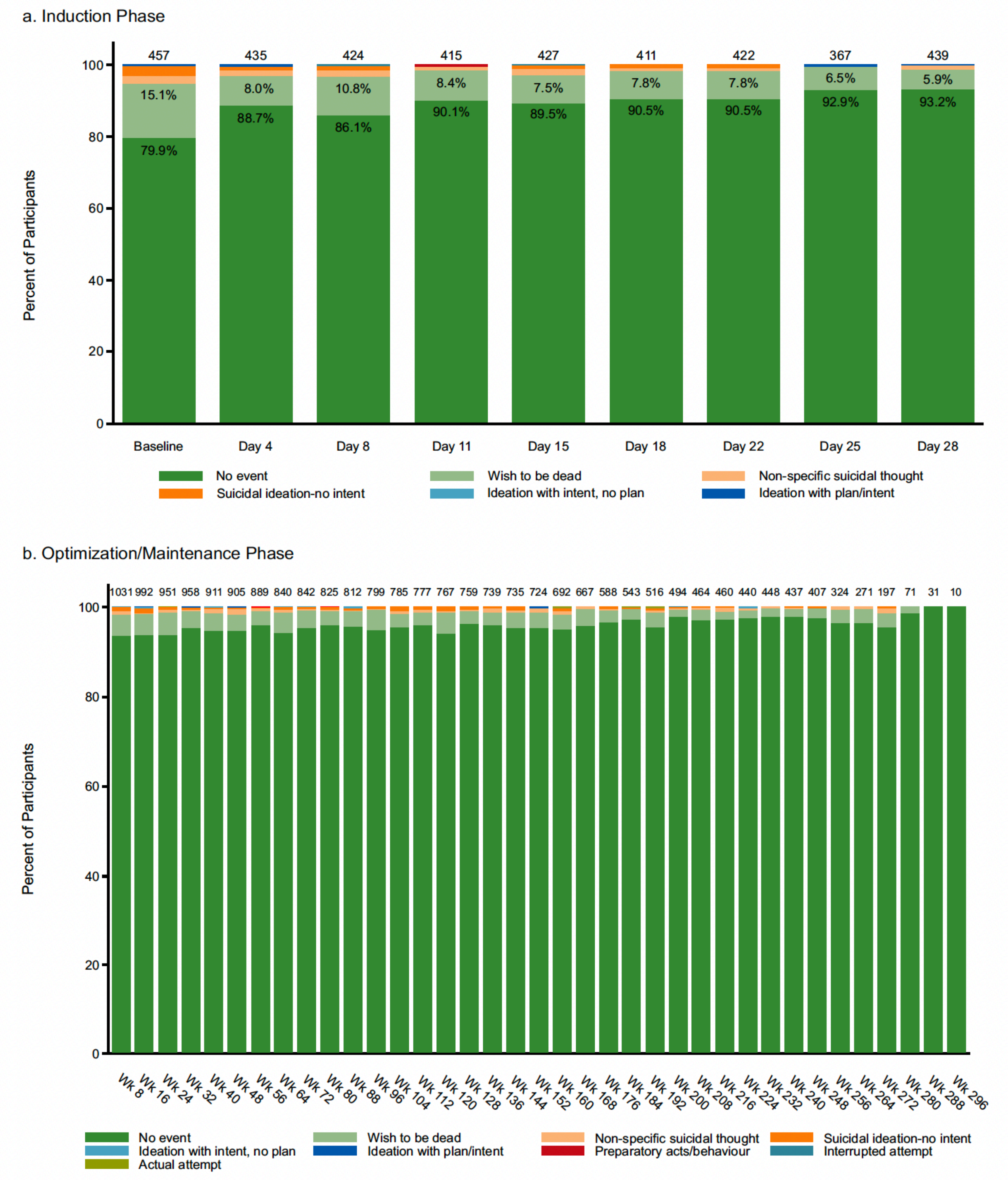


Note: Visits with the number of participants <10 are not presented.

**Figure S17. Mean (± SE) Patient Health Questionnaire 9-Item (PHQ-9) Total Score (Observed Cases)**

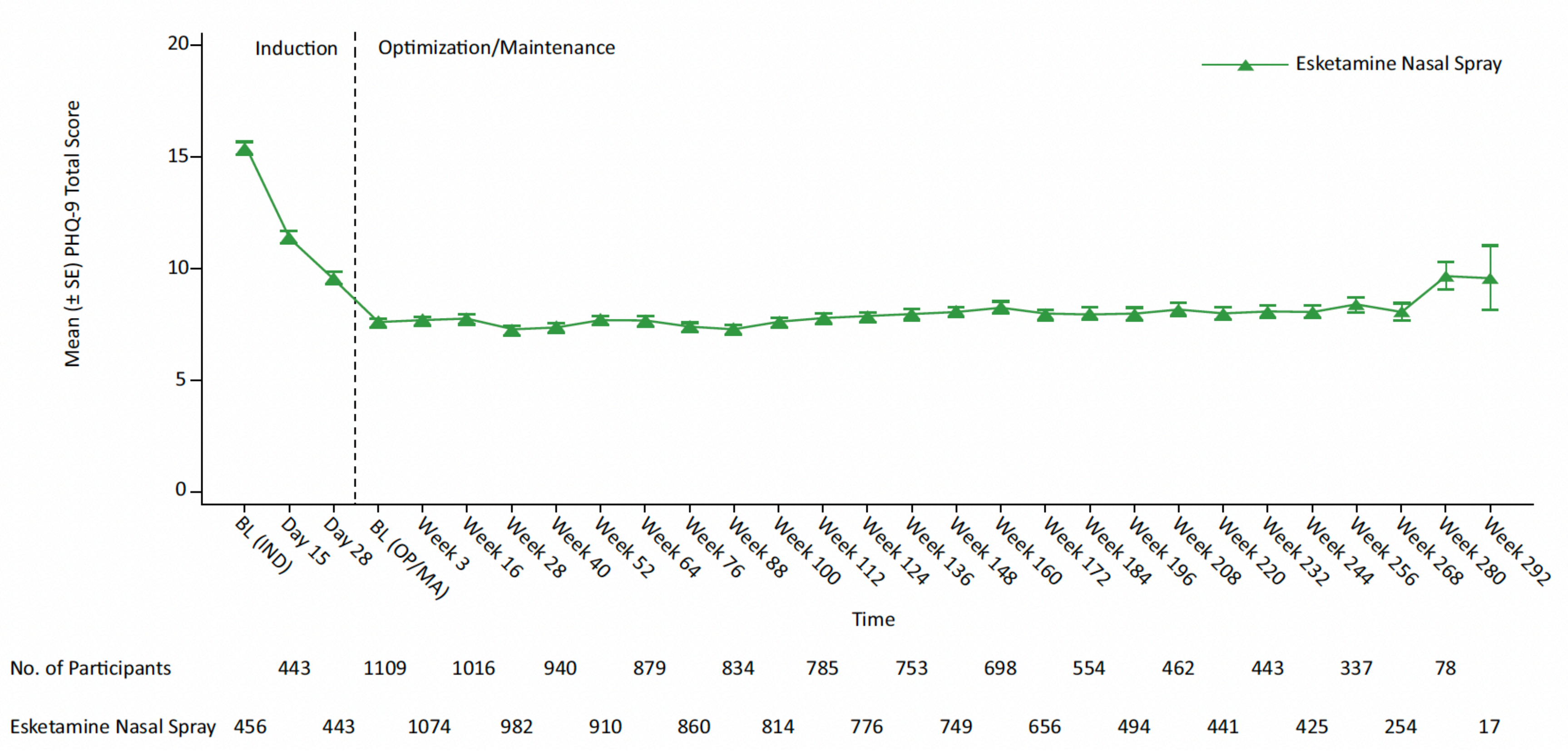


BL (IND) = baseline (induction phase); BL (OP/MA) = baseline (optimization/maintenance phase); PHQ = Patient Health Questionnaire; SE = standard error. Data for the induction phase are reported elsewhere [Zaki et al., 2023].

Zaki N, Chen LN, Lane R, et al. Long-term safety and maintenance of response with esketamine nasal spray in participants with treatment-resistant depression: interim results of the SUSTAIN-3 study. Neuropsychopharmacology. 2023;48(8):1225-1233. doi: 10.1038/s41386-023-01577-5

**Figure S18. Mean (± SE) Sheehan Disability Scale Total Score (Observed Cases)**

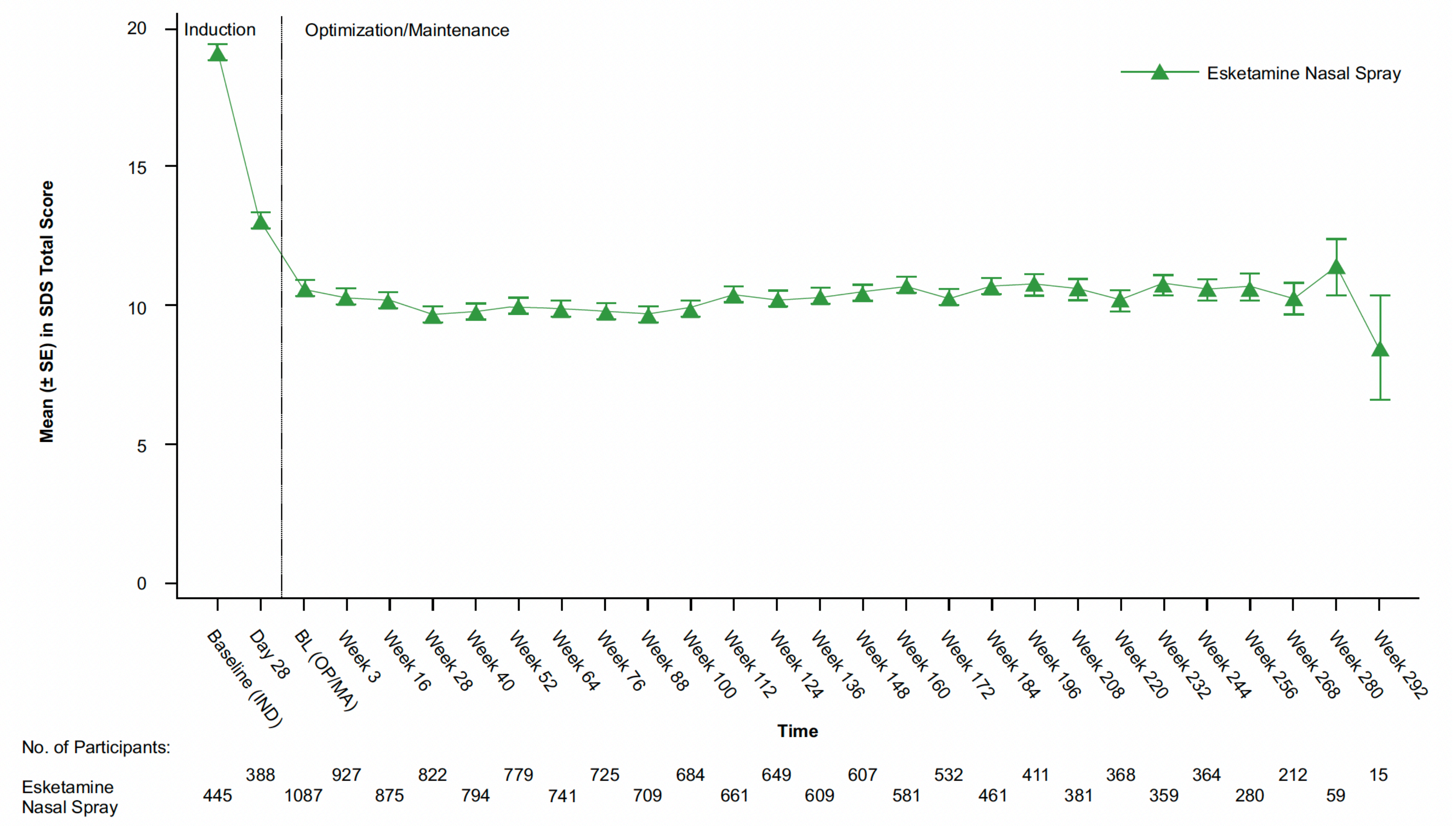


IND = induction phase; OP/MA = optimization/maintenance phase; SDS = Sheehan Disability Scale; SE = standard error.
